# TRIM65 promotes renal cell carcinoma through ubiquitination and degradation of BTG3

**DOI:** 10.1038/s41419-024-06741-3

**Published:** 2024-05-22

**Authors:** Qi Zhang, Yong Li, Qing Zhu, Tao Xie, Yue Xiao, Feng Zhang, Na Li, Keyu Deng, Hongbo Xin, Xuan Huang

**Affiliations:** 1 The National Engineering Research Center for Bioengineering Drugs and the Technologies, Institute of Translational Medicine, Jiangxi Medical College, Nanchang University, Nanchang, 330031 China; 2 Department of Anesthesiology, The First Affiliated Hospital, Jiangxi Medical College, Nanchang University, Nanchang, 330006 China; 3 First School of Clinical Medicine, Jiangxi Medical College, Nanchang University, Nanchang, 330031 China; 4 School of Future Technology, Nanchang University, Nanchang, 330031 China

**Keywords:** Ubiquitylation, Oncogenes, Renal cell carcinoma, Oncogenesis

## Abstract

As a typical E3 ligase, TRIM65 (tripartite motif containing 65) is involved in the regulation of antiviral innate immunity and the pathogenesis of certain tumors. However, the role of TRIM65 in renal cell carcinoma (RCC) and the underlying mechanism has not been determined yet. In this study, we identified TRIM65 as a novel oncogene in RCC, which enhanced the tumor cell proliferation and anchorage-independent growth abilities both in vitro and in vivo. Moreover, we found that TRIM65-regulated RCC proliferation mainly via direct interaction with BTG3 (BTG anti-proliferation factor 3), which in turn induced the K48-linked ubiquitination and subsequent degradation through K41 amino acid. Furthermore, TRIM65 relieved G2/M phase cell cycle arrest via degradation of BTG3 and regulated downstream factors. Further studies revealed that TRIM65 acts through TRIM65-BTG3-CyclinD1 axis and clinical sample IHC chip data indicated a negative correction between TRIM65 and BTG3. Taken together, our findings demonstrated that TRIM65 promotes RCC cell proliferation via regulation of the cell cycle through degradation of BTG3, suggesting that TRIM65 may be a promising target for RCC therapy.

## Introduction

Renal cell carcinoma (RCC), accounting for 2–3% of adult malignant tumors, is the most lethal neoplasm of the genitourinary system, which is more common in males [1]. In recent decades, the incidence rate of RCC has increased steadily in most parts of the world [2]. In China, RCC ranks second in the incidence rate of urogenital tumors, only after bladder cancer [3]. RCC can be divided into three main subtypes: clear cell RCC (ccRCC), papillary RCC (pRCC), and chromophobe RCC (chRCC). The most prevalent subtype, ccRCC, accounts for around 75% of all RCC cases [4]. About 40% of RCC patients still suffer from tumor recurrence and metastasis, which can ultimately lead to death, despite the fact that clinical treatment of RCC has developed significantly over the past ten years and the new surgery & systematic treatments have obviously improved the prognosis [5]. Sunitinib is the only one systemic adjuvant drug approved by FDA for the treatment of RCC so far in China, which can merely prolong the progression-free survival for two months with severe drug-related toxicity [6]. Therefore, exploring new therapeutic targets is helpful in developing new anticancer drugs and improving the efficacy of RCC patients.

The ubiquitin-mediated protein degradation pathways play important roles in the elimination of transient regulatory proteins, involved in cell cycle regulation, DNA repair, morphogenesis, protein quality control, and so on [7]. Almost all oncogenes and tumor suppressor genes are regulated by post-translational modifications, including ubiquitin-proteasome system [8]. Therefore, the identification and targeting of E3 ligases related to the regulation of oncoproteins and tumor suppressor proteins has become the focus of tumor research. Recent studies have shown that E3 ubiquitin ligases were involved in the occurrence and development of RCC [9], such as FBXW7. Tumor suppressor FBXW7 inhibited the proliferation, migration and invasion of ccRCC cells via ubiquitination and degradation of MLST8 [10]. A pan-cancer analysis showed that E3 ubiquitin ligase NEDD4L was lower expressed in ccRCC tissues, and NEDD4L may suppress tumor cell proliferation and migration by inhibiting ERBB3 and MAPK signaling pathways [11]. Additionally, an increasing number of studies have verified that members of E3 ligase tripartite motif (TRIM) family proteins can also participate in the regulation of RCC. TRIM37 promoted the EMT and malignant behavior of RCC cells via H2A ubiquitination, activating the TGF-β1 signaling pathway [12]. TRIM27 facilitated the proliferation of RCC cells through the NF-κB signaling pathway [13]. TRIM47 promoted the malignant progression of RCC through ubiquitination and degradation of p53 [14], while TRIM44 can accelerate RCC cell proliferation and migration by inhibiting FRK [15]. However, the majority of the mechanisms remain unclear and there is no further research on the transformation and application of E3 ubiquitin ligases’ function.

As a member of the TRIM family, TRIM65 is a typical E3 ligase with “RBCC” domain (an N-terminal RING domain, a B-box, a coiled-coil, and a C-terminal SPRY domain). The research on the function of TRIM65 began in 2011, GWAS results suggest that the SNP of *TRIM65* gene may be relevant to white matter lesions [16]. Subsequently, Li et al. found that TRIM65 can degrade TNRC6 through ubiquitination and participate in the functional regulation of miRNA [17]. Besides, TRIM65 also plays a critical role in antiviral innate immunity [18, 19]. As an E3 ubiquitin ligase, TRIM65 promotes the occurrence and development of a variety of tumors by degrading tumor-related proteins, such as ARHGAP35 [20], ANXA2 [21], Axin1 [22], p53 [23], PPM1A [24], etc. However, the research on the function and mechanism of TRIM65 is still in its infancy, and the role of TRIM65 in RCC is never reported.

Here we identified TRIM65 as a new E3 ubiquitin ligase of BTG3. BTG3 is a member of the BTG/Tob family, with a conserved N-terminal sequence of 104–106 amino acids [25]. BTG family proteins generally inhibit cell proliferation and can also participate in cell cycle progression and differentiation of different kinds of cells. BTG3 has been reported to be involved in the occurrence and development of many tumors as a tumor suppressor [26, 27], such as ovarian cancer, gastric cancer, lung cancer, etc. Although BTG3 was validated to be a transcriptional target of p53 [28], the post-translational modification of BTG3 has never been reported.

In our study, we found that TRIM65 is highly expressed in clinical RCC tissues and cell lines. Downregulation of TRIM65 can significantly suppress cell proliferation both in vitro and in vivo while TRIM65 upregulation promoted cell proliferation. Furthermore, we found that TRIM65 regulated RCC proliferation mainly through direct ubiquitination with BTG3. IHC chip data of clinical samples revealed that the expression levels of TRIM65 and BTG3 were negatively correlated. These results suggest that TRIM65 functioned as a novel oncogene through ubiquitination and degradation of BTG3.

## Results

### The expression of TRIM65 was elevated in human RCC tissues

To explore the role of TRIM65 in RCC, we first analyzed the mRNA expression of TRIM65 in renal cancer patients from the TCGA database via the UALCAN portal. Compared to adjacent normal tissues, TRIM65 was highly expressed in renal clear cell carcinoma samples (Fig. S1A). As shown in Fig. S1B, TRIM65 mRNA levels increased with each stage, except for stage II. It’s interesting to note that the amount of TRIM65 was positively related to the lymph node metastasis of patients (Fig. S1C). Moreover, ccRCC patients with high TRIM65 levels (*n* = 261) had a worse chance of survival and shorter living periods than those with low TRIM65 expression (*n* = 261, Fig. S1D). Consistently, the protein levels of TRIM65 were elevated significantly in ccRCC tissues as well (Fig. S1E–G). These results suggest that TRIM65 was upregulated in RCC tissue samples, which may play important roles in the occurrence and development of RCC.

### Knockdown of TRIM65 suppressed the proliferation of RCC cells

**Fig. 1:**
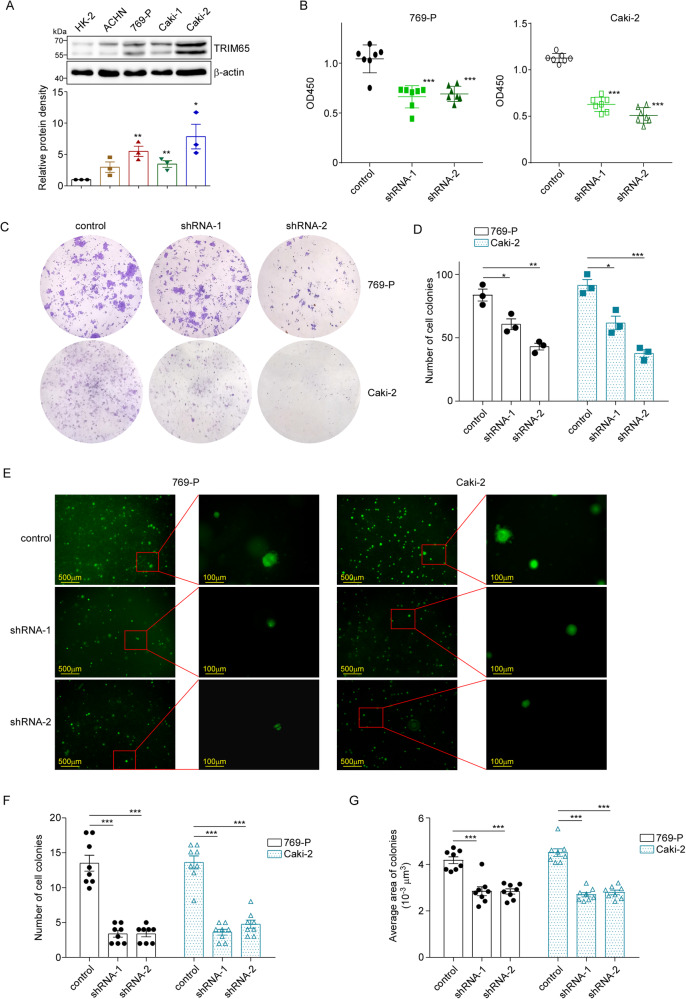
Interference with TRIM65 inhibited the proliferation of renal cell carcinoma cells. **A** The expression of TRIM65 protein in four renal cell carcinoma cell lines and control HK-2 cells. **B** CCK8 assay. Cells were infected with lentivirus TRIM65 shRNA-1/shRNA-2 or control to generate stable cell lines. Then CCK8 cell proliferation assay was performed using those stable cells. **C** Colony formation assay. 10–14 days later, the indicated cells grown in 6-well plates were fixed and stained with 2% crystal violet. **D** The number of colonies was counted and data are presented as mean ± SEM (*n* = 3). **E** Soft agar colony formation assay. The above stable RCC cells were seeded in 6-well plates. 15–20 days later, cells were imaged, colonies larger than 50 μm were counted, and calculated the area. **F** The number of cell colonies was calculated in eight randomly selected views. Data are presented as mean ± SEM (*n* = 8). **G** The average area of colonies was calculated in eight randomly selected views. Data are presented as mean ± SEM (*n* = 8). *p < 0.05, **p < 0.01, ***p < 0.001 by Student’s *t*-test.

Next, we investigated the role of TRIM65 in the proliferation and anchorage-independent growth of RCC cells in vitro. Firstly, we examined the expression of TRIM65 in RCC cell lines including ACHN, 769-P, Caki-1, and Caki-2, and HK-2, a renal tubular epithelial cell line was used as normal control. Results of western blotting (Fig. 1A) showed that all renal cancer cells had significantly raised levels of TRIM65 protein. Consistently, most cancer cell lines had elevated transcription levels with the exception of ACHN cells (Fig. S2A). To explore its biological function, we knocked down TRIM65 in 769-P/Caki-2 cell lines with relative higher TRIM65 protein levels (Fig. S2D, E). CCK8 results showed that stable transfection of different shRNAs (shRNA-1 and shRNA-2) can inhibit the proliferation of two cell lines tested (Fig. 1B). Accordingly, TRIM65 knockdown suppressed RCC cell growth in full growth medium (Fig. S3A) and low serum medium (Fig. S3B). As expected, downregulation of TRIM65 also repressed the colony formation of cancer cells (Fig. 1C, D). Furthermore, we found that knockdown of TRIM65 obviously weakened the anchorage-independent growth abilities of renal cancer cells through soft agar assays (Fig. 1E–G).

### Upregulation of TRIM65 promoted the proliferation of RCC cells

To confirm the role of TRIM65 in RCC cell proliferation, we established TRIM65 overexpression-stable cells in ACHN and Caki-1 cell lines with relative lower TRIM65 protein levels (Fig. S2B, C). Overexpression of TRIM65 significantly increased the proliferation of renal cancer cells (Fig. S4A), as well as the RCC cell growth both in low serum medium (Fig. S4B) and full growth medium (Fig. S4C). Moreover, upregulation of TRIM65 promoted the colony formation (Fig. S4D) and anchorage-independent growth (Fig. S4E–G) of renal cancer cells, which were consistent with the results of TRIM65 knockdown. Taken together, TRIM65 dramatically promotes the proliferation of RCC cells.

### TRIM65 promoted tumor growth in vivo

**Fig. 2:**
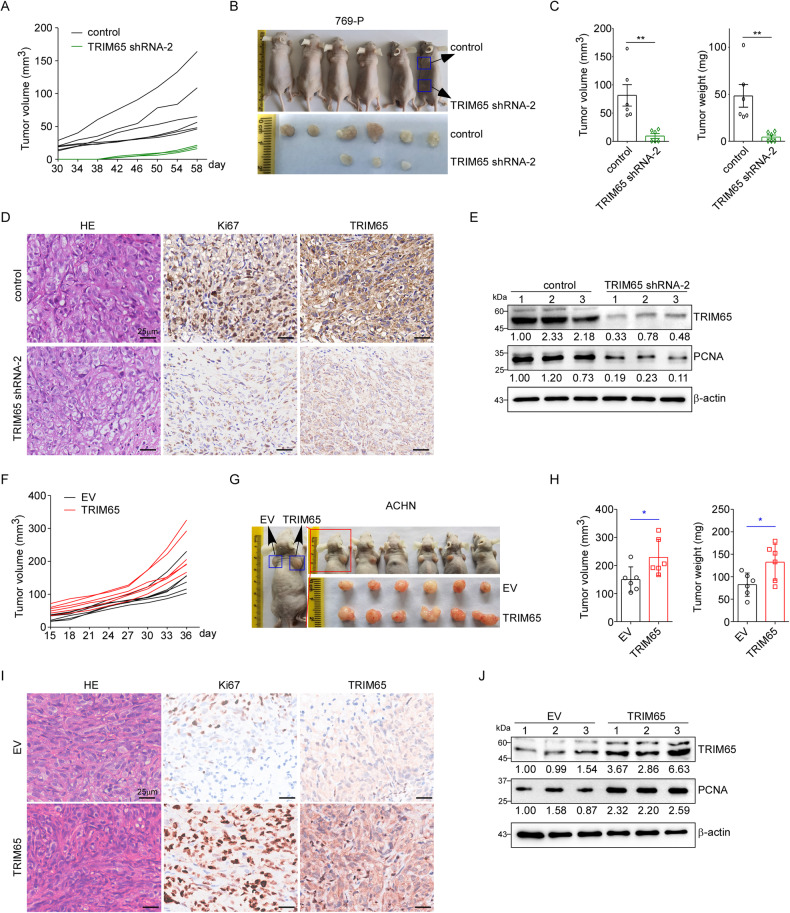
TRIM65 promoted tumor growth of RCC cells in vivo. **A**–**C** TRIM65 knockdown inhibited the formation of tumors with 769-P cells in vivo. Six nude mice were injected subcutaneously with the above stable cell lines. 30 days later, tumor volume was measured every four days (**A**). Tumors were isolated, measured, and weighed. The images of mice and tumors were shown in (**B**), while data of the tumor volume and weight were listed in (**C**). Data was shown as mean ± SEM (*n* = 6). **p < 0.01. **D** Downregulation of TRIM65 suppressed the proliferation of tumor cells. Tumors were isolated, fixed, and employed to HE staining and IHC assays. **E** TRIM65 knockdown reduced the protein levels of PCNA in vivo. **F**–**H** Overexpression of TRIM65 promotes ACHN cell growth in a xenograft model. Six nude mice were injected subcutaneously with the indicated cells. 15 days later, tumor volume was measured every three days (**F**). The images of mice and tumors were shown in (**G**), while data of the tumor volume and weight were listed in (**H**). Data was shown as mean ± SEM (*n* = 6). *p < 0.05. **I** TRIM65 promoted the proliferation of tumor cells detected by HE and IHC assays. **J** Upregulation of TRIM65 increased the protein expression of PCNA in vivo.

To determine the effect of TRIM65 on RCC tumor growth in a more significant context, we then performed xenograft experiments using 769-P and ACHN cells in nude mice. Results of Fig. 2A–C indicated that downregulation of TRIM65 in 769-P cells significantly suppressed both the average tumor volume and tumor weight. As shown in the results of IHC (Fig. 2D) and western blotting (Fig. 2E), the decreased expression of proliferation markers, Ki67 and PCNA, was in line with the above results, demonstrating that TRIM65 knockdown inhibited tumor growth in vivo. In contrast, overexpression of TRIM65 resulted in a dramatic increase in both the volume and weight of tumors using ACHN cells compared to the control group (Fig. 2F–H). Upregulation of TRIM65 resulted in higher protein levels of Ki67 and PCNA as well (Fig. 2I, J). The collective results proved that TRIM65 plays a key role in facilitating the growth of xenograft tumors.

### TRIM65 directly interacted with BTG3

**Fig. 3:**
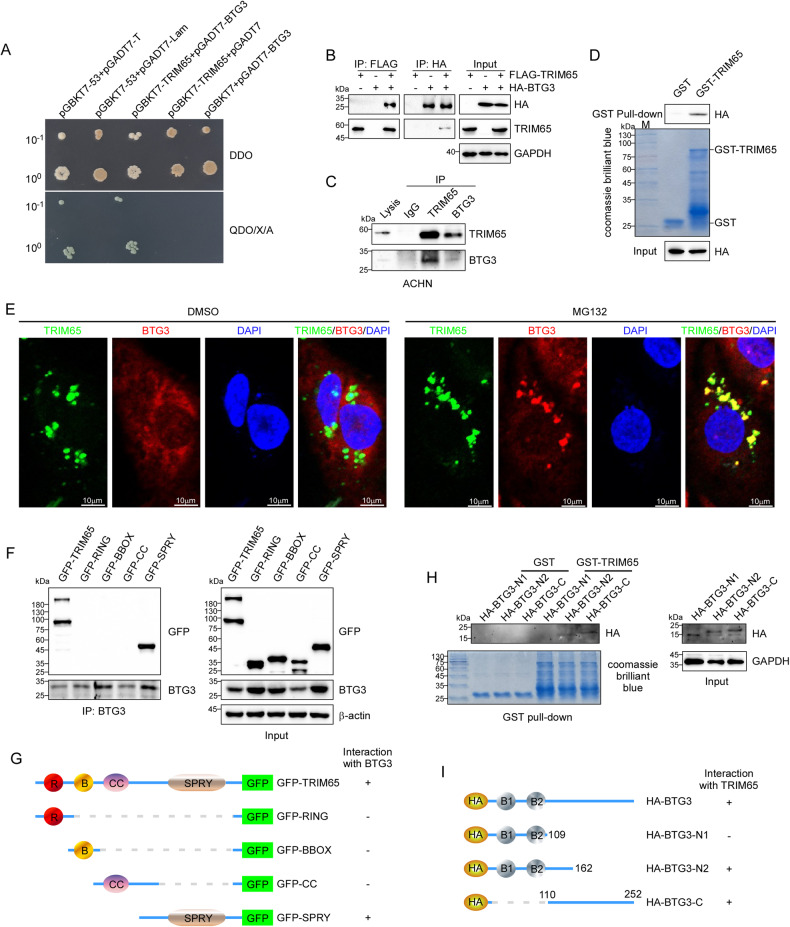
TRIM65 interacted with BTG3. **A** BTG3 was identified as a new binding protein of TRIM65 via the yeast-two-hybrid system. **B** Co-immunoprecipitation analysis of the interaction between FLAG-TRIM65 and HA-BTG3. **C** Endogenous interaction of TRIM65 and BTG3. **D** The purified GST or GST-TRIM65 protein was incubated with the lysates of BTG3-transfected HEK293T cells. **E** Co-localization of TRIM65 and BTG3 in RCC cells. ACHN cells were co-stained with antibodies against BTG3 and TRIM65 with or without MG132. **F** Interaction of BTG3 with GFP-TRIM65 and its deletion mutants was determined. **G** Schematic diagram of TRIM65 deletion mutants generated in pEGFP-C vectors. **H** Interaction of TRIM65 with BTG3 and its deletion mutants was determined by GST pull-down and western blotting analysis in HEK293T cells transfected with BTG3 and its deletion mutants**. I** Schematic diagram of BTG3 deletion mutants generated in pCMV-HA vectors.

As we have confirmed that TRIM65 functioned as an oncogene in RCC, we then use full-length human TRIM65 protein as the bait in a yeast-two-hybrid assay to screen for the interacting protein candidates and explore the underlying mechanism. BTG3, a member of the BTG/Tob family, was isolated as the binding protein, the interaction between BTG3 and TRIM65 was further validated using the yeast hybrid assay (Fig. 3A). Next, the binding between these two proteins was confirmed by a co-immunoprecipitation assay from lysates of HEK293T cells expressing both proteins (Fig. 3B). The endogenous interaction between TRIM65 and BTG3 was also performed in ACHN cells using specific TRIM65 and BTG3 antibodies (Fig. 3C). To further validate that TRIM65 physically interacts with BTG3, we performed GST pull-down assays and found that BTG3 can be pulled down by GST-TRIM65 but not GST (Fig. 3D). Interestingly, overexpression of TRIM65 could degrade the exogenous BTG3 and no/weak co-localization of ectopic TRIM65 with BTG3 was observed (Fig. 3E left). Moreover, the co-localization of exogenous FLAG-TRIM65 and HA-BTG3 was enhanced and can be visualized easily after MG132 treatment (Fig. 3E right), indicating that TRIM65 may target BTG3 for proteasome-dependent degradation. Also, the localization of BTG3 was almost overlapped with that of TRIM65 only after MG132 treatment.

Based on the structure characteristics of TRIM65 and BTG3, we established a series of plasmids with various domain mutant (Fig. 3G, I) to identify the specific binding sequences. Firstly, Co-IP assays were carried out to confirm the binding domain of TRIM65 following MG132 treatment. Besides full-length TRIM65, only the SPRY domain (GFP-SPRY) can be co-immunoprecipitated with BTG3, showing that BTG3 binds to the SPRY domain of TRIM65 (Fig. 3F). As for BTG3, both the N2 and C fragments of BTG3 can be pulled down by GST-TRIM65 but not the N1 fragment, suggesting that TRIM65 may interact with the 110aa-162aa fragment of BTG3 (Fig. 3H). These results showed that TRIM65 directly interacted with BTG3 (110aa-162aa) via the SPRY domain.

### TRIM65 promoted BTG3 degradation via K48-linked ubiquitination

**Fig. 4:**
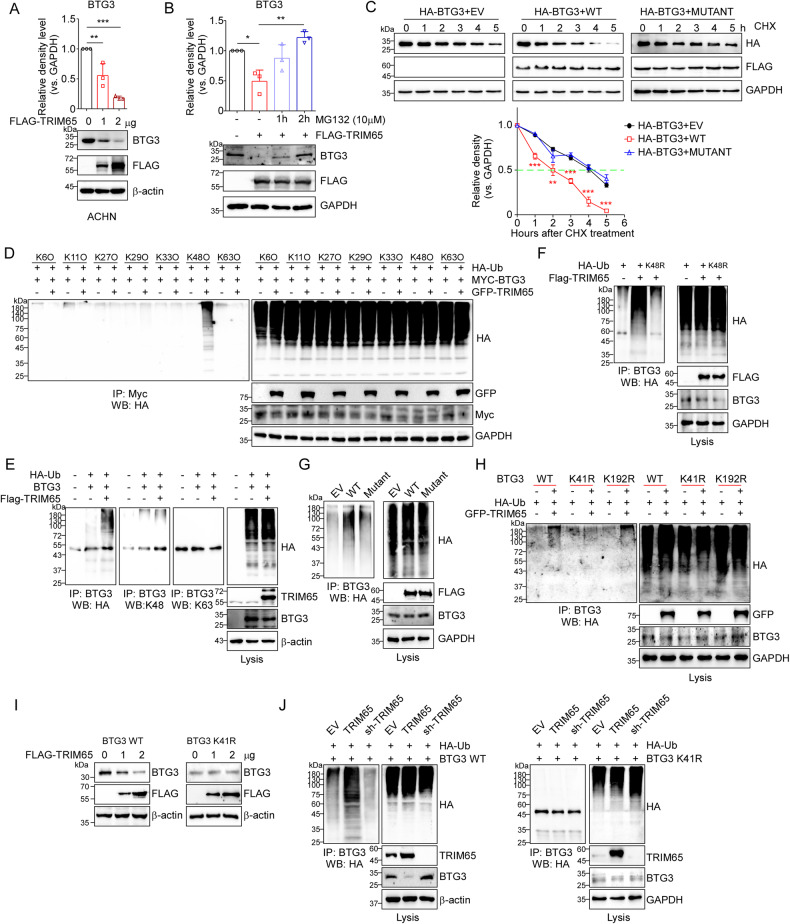
TRIM65 promoted K48-linked ubiquitination of BTG3 and its degradation. **A** Overexpression of TRIM65 suppressed the protein levels of BTG3 in a dose-dependent manner. **B** MG132 can restore the protein decline of BTG3 induced by TRIM65. **C** Overexpression of TRIM65 but not TRIM65 mutant accelerated the degradation of BTG3 protein. HEK293T cells expressing EV or TRIM65 or TRIM65 mutant were treated with cycloheximide (CHX) for indicated times. Data was shown as mean ± SEM (*n* = 3). **p < 0.01, ***p < 0.001. **D** HEK293T cells were co-transfected with BTG3 plus HA-Ub mutants as indicated (e.g. K6O means K6 only) and/or GFP-TRIM65. **E** HEK293T cells were co-transfected with BTG3 plus HA-ubiquitin (HA-Ub) and/or FLAG-TRIM65. **F** BTG3 and FLAG-TRIM65 were co-transfected with HA-Ub or HA-Ub K48R into HEK293T cells. **G** HEK293T cells were co-transfected with BTG3 and EV, FLAG-TRIM65 (WT) or FLAG-TRIM65 mutant (Mutant). **H** HEK293T cells were co-transfected with HA-Ub and GFP-TRIM65 plus BTG3 (WT), BTG3 K41R, or BTG3-K192R. Cell lysates were harvested and immunoprecipitated with anti-BTG3 (anti-Myc) antibody, and were also detected using the indicated antibodies by western blotting (**D**–**H**). **I** Ectopic transfection of TRIM65 can only modulate the protein levels of BTG3 WT, but not BTG3 K41R. **J** Upregulation or knockdown of TRIM65 cannot affect the ubiquitination levels of BTG3 K41R.

TRIM65 has been reported and identified as an E3 ubiquitin ligase in previous studies [29, 30], so we intended to explore the effect of TRIM65 on the protein levels of BTG3 and the molecular mechanism. As shown in Fig. 4A, BTG3 protein levels were dose-dependently down-regulated by TRIM65 overexpression. The proteasome inhibitor MG132 can significantly stabilize BTG3 when TRIM65 was transiently transfected in HEK293T cells, indicating that BTG3 may be subjected to the modulation of ubiquitin-proteasome system via TRIM65 (Fig. 4B). Moreover, transfection of TRIM65 shortened the half-life of BTG3 to 2 h (50% of control plasmid), whereas the TRIM65 RING mutant which cannot bind to E2 (E3 ligase lost-of-function mutant), has almost no effect (Fig. 4C). These collective results suggest that BTG3 may be a substrate of TRIM65, which can be degraded via TRIM65 in a UPS dependent way. Next, we figured out whether and how TRIM65 controlled the ubiquitination of BTG3. Firstly, we explored the lysine linkage way of ubiquitination of BTG3 via seven different Ub mutants including, K6O, K11O, K27O, K29O, K33O, K48O, and K63O, as indicated in Fig. 4D, revealing that TRIM65 promoted K48-linked ubiquitination of BTG3. We then verified that ectopic expression of TRIM65 enhanced BTG3 ubiquitination in HEK293T cells in a K48-linked way but not K63 linkage (Fig. 4E). In addition, HA-Ub K48R mutant cannot be linked to BTG3 with TRIM65 overexpression, which further confirmed that TRIM65 can only promote K48-linked ubiquitination of BTG3 (Fig. 4F). Besides, the RING finger domain of TRIM65 was indispensable for the BTG3 ubiquitination since the TRIM65 RING domain mutant (Mutant) cannot induce BTG3 ubiquitination (Fig. 4G).

The ubiquitination modification of BTG3 has not been reported yet, we then further identified its specific ubiquitination site mediated by TRIM65. Based on the alignment of the BTG3 protein sequences from different species, we obtained the conserved lysine sites of BTG3 which were shown in Fig. S5A. Then, we generated a series of Myc-BTG3 plasmids carrying different lysine-to-arginine mutations and co-transfected each BTG3 plasmid into HEK293T cells together with either GFP-TRIM65 or GFP control vector. We found that two mutations, K41R and K192R, appear to be important for TRIM65-mediated BTG3 ubiquitination and degradation following the first round of screening using western blotting assays (Fig. S5B). Further ubiquitination results (Fig. 4H) showed that TRIM65 had no effect on the ubiquitination of K41R mutant BTG3, indicating that TRIM65-mediated K48-linked ubiquitination of BTG3 through lysine 41(K41). Consistently, overexpression of TRIM65 can only regulate the protein levels of BTG3 WT but not BTG3 K41R (Fig. 4I). Besides, upregulation or knockdown of TRIM65 cannot change the ubiquitination levels of BTG3 K41R, compared to BTG3 WT counterpart (Fig. 4J). In summary, TRIM65 can promote the K48-linked ubiquitination of BTG3 at K41 for its subsequent degradation.

### TRIM65 relieved G2/M phase cell cycle arrest via BTG3

**Fig. 5:**
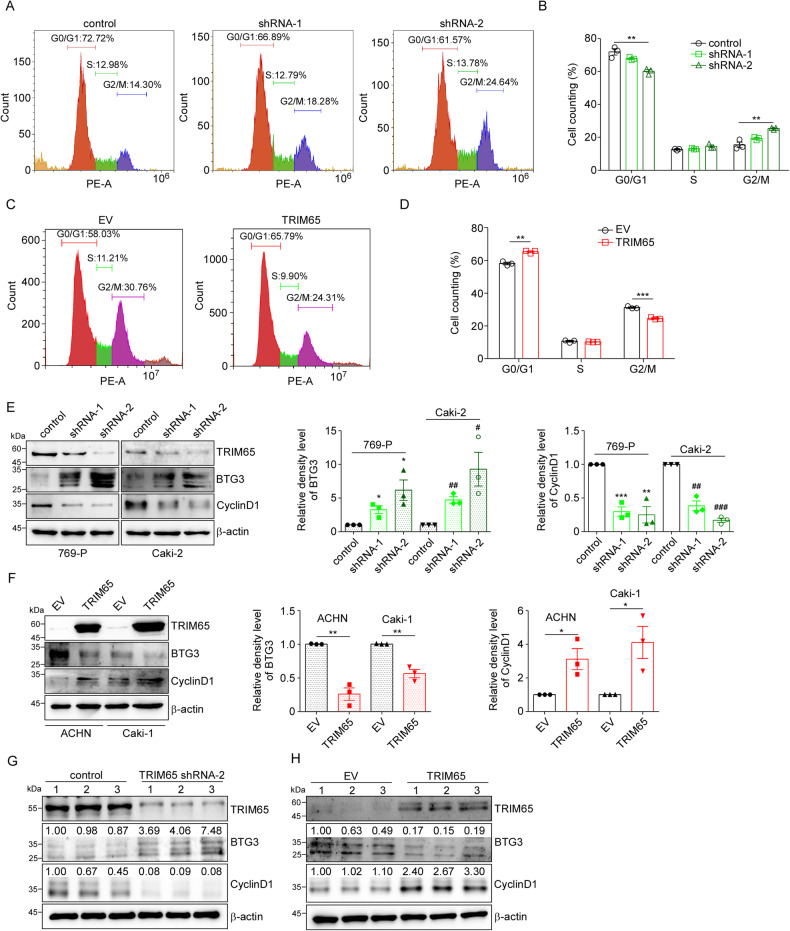
TRIM65 relieved G2/M phase cell cycle arrest via BTG3. **A**, **B** Cell cycle analysis of 769-P cells stably knockdown of TRIM65 or control via flow cytometry after PI staining. **C**, **D** Cell cycle analysis of ACHN cells stably overexpressing of TRIM65 or EV. Data was shown as mean ± SEM (*n* = 3). **p < 0.01, ***p < 0.001. **E** Downregulation of TRIM65 enhanced the protein levels of BTG3 and suppressed the expression of CyclinD1. *p < 0.05, **p < 0.01, ***p < 0.001, compared with 769-P control group; #*p* < 0.05, ##*p* < 0.01, ###*p* < 0.001, compared with Caki-2 control group. **F** Upregulation of TRIM65 reduced the protein levels of BTG3 while raised the CyclinD1 proteins levels. *p < 0.05, **p < 0.01. **G** Knockdown of TRIM65 in 769-P cells enhanced the protein levels of BTG3 in the tumors of xenograft models. **H** Overexpression of TRIM65 in ACHN cells inhibited the protein expression of BTG3 in the tumors of xenograft models.

BTG3 is known as a tumor suppressor in many kinds of cancers, BTG3 knockdown could relieve G2 phase arrest in colorectal cancer cells [31]. Due to the regulatory effect of TRIM65 on BTG3, we then explored the role of TRIM65 in the cell cycle. Consistent with the cell proliferation studies, TRIM65 knockdown induced cell cycle arrest at G2/M phase with elevated percentage of G2/M phase cells (Fig. 5A, B), whereas overexpression of TRIM65 significantly increased the proportions of cells in G0/G1 phase, thus relieved G2 phase arrest (Fig. 5C, D). BTG3 plays a key role in cell cycle progression by regulating cell cycle-dependent expression of genes important for cell growth, for example, CyclinD1, p21, p27, etc. As shown in Fig. 5E, knockdown of TRIM65 increased the protein levels of BTG3, which was consistent with TRIM65 overexpression results (Fig. 5F), indicating that TRIM65 may regulate cell cycle progression via BTG3. After qRT-PCR screening and western blotting confirmation (data not shown), we found that TRIM65 promotes the transcription of CyclinD1 but not p21 and p27 (Fig. 5E, F). Furthermore, we detected the indicated protein levels in tumors from xenograft models (Fig. 5G, H) and found that TRIM65 promoted the proliferation of tumor cells via TRIM65-BTG3-CyclinD1 axis. Results of IHC staining of BTG3 were consistent with the above western blotting data (Fig. S6A).

### The function of TRIM65 on RCC cell proliferation is dependent on BTG3

**Fig. 6:**
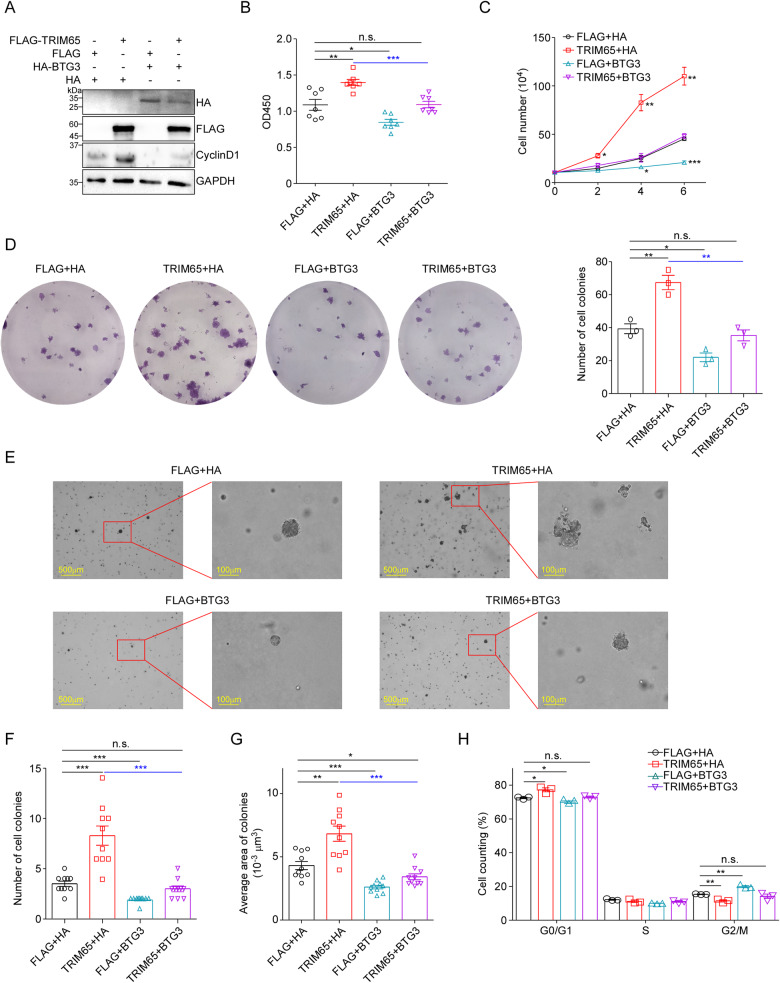
The function of TRIM65 on RCC cell proliferation is dependent on BTG3. **A** Western blotting analysis of 4 groups for co-transfection into ACHN cells as indicated. **B** CCK8 assay. Cells were co-transfected with the indicated vectors, then subjected to CCK8 analysis. **C** Low serum assay. At the indicated times, 4 groups of transfected ACHN cells were trypsinized and counted. Data are presented as mean ± SEM (*n* = 3). **D** Colony formation assay. After 10–14 days, cells of each group were fixed and stained with 2% crystal violet. The number of cell colonies were calculated. **E** Soft agar colony formation assay. 4 groups of ACHN cells were seeded in 6-well plates. 15–20 days later, cells were imaged, colonies larger than 50 μm were counted, and calculated the area. **F** The number of cell colonies were calculated in ten random selected views. Data are presented as mean ± SEM (*n* = 10). **G** The average area of colonies was calculated in ten random selected views. **H** Cell cycle analysis of indicated ACHN cells via flow cytometry after PI staining. Data are presented as mean ± SEM (*n* = 10). n.s. *p* > 0.05, *p < 0.05, **p < 0.01, ***p < 0.001 by Student’s *t*-test.

To verify that the regulation of TRIM65 on the proliferation of RCC cells is through BTG3 degradation, we set up 4 groups for transfection into ACHN cells as indicated in Fig. 6A. Our results showed that TRIM65 overexpression increased the proliferation of ACHN cells which was markedly blocked by the co-transfection of BTG3 (Fig. 6B, C). Similarly, supplement with BTG3 protein suppressed the colony formation of cancer cells elevated by TRIM65 overexpression (Fig. 6D). Furthermore, we found that ectopic overexpression of BTG3 eliminated the acceleration of TRIM65 on the anchorage-independent growth abilities of renal cancer cells in soft agar assays (Fig. 6E–G). Meanwhile, BTG3 replenishment via transient transfection can significantly release the cell cycle arrest induced by TRIM65 overexpression (Fig. 6H). Taken together, TRIM65 facilitated the proliferation of RCC cells dependent on BTG3.

### TRIM65 cannot modulate the function of BTG3 K41R

**Fig. 7:**
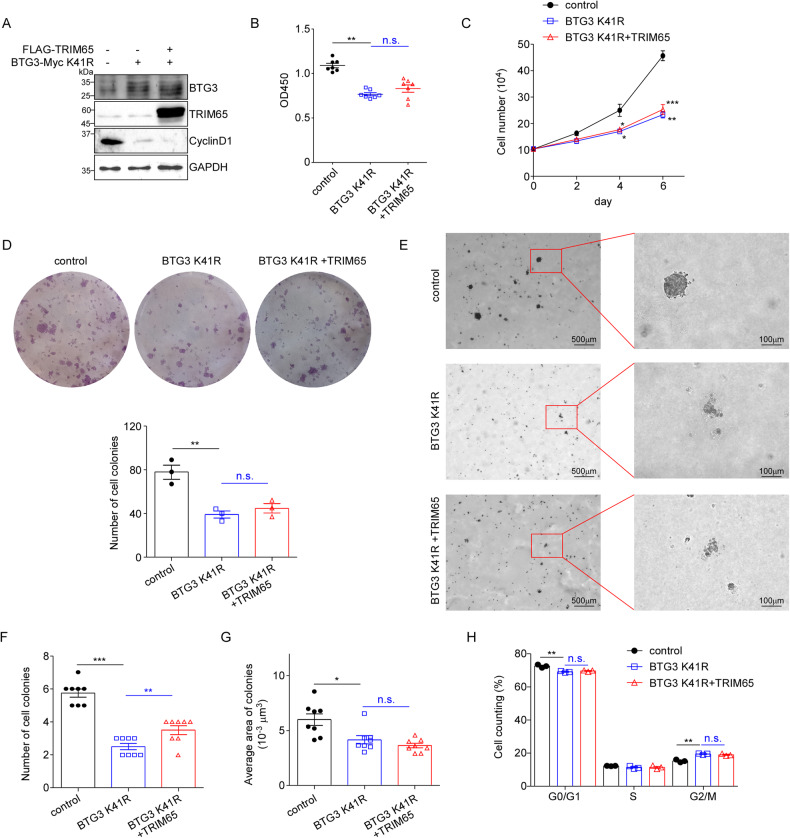
TRIM65 functions as an oncoprotein through K41 ubiquitination of BTG3. **A** Western blotting analysis of 3 groups for transfection into ACHN cells. **B** CCK8 assay. Cells were transfected with the indicated vectors, then subjected to CCK8 analysis. **C** Low serum assay. At the indicated times, cells were trypsinized and counted. Data are presented as mean ± SEM (*n* = 3). **D** Colony formation assay. After 10–14 days, cells grown in 6-well plates were fixed and stained with 2% crystal violet. The number of cell colonies were calculated. **E** Soft agar colony formation assay. 3 groups of ACHN cells were seeded in 6-well plates. 15–20 days later, cells were imaged, colonies larger than 50 μm were counted, and calculated the area. **F** The number of cell colonies were calculated in eight random selected views. Data are presented as mean ± SEM (*n* = 8). **G** The average area of colonies was calculated in eight random selected views. **H** Cell cycle analysis of indicated ACHN cells via flow cytometry after PI staining. Data are presented as mean ± SEM (*n* = 8). n.s. *p* > 0.05, *p < 0.05, **p < 0.01, ***p < 0.001 by Student’s *t*-test.

Since BTG K41 was validated to be responsible for TRIM65-mediated ubiquitination, we then investigated the effect of this mutant on the growth of ACHN cells. As shown in Fig. 7A–D, overexpression of BTG3 K41R alone inhibited the proliferation and colony formation of cancer cells, indicating that the K41R mutation didn’t affect the anti-tumor effect of BTG3. As expected, co-transfection with TRIM65 cannot neutralize the effect of BTG3 K41R on the growth of RCC cells. Meanwhile, upregulation of BTG3 K41R also suppressed the anchorage-independent growth of ACHN cells, while co-transfection of TRIM65 cannot affect the ability of anchorage-independent growth (Fig. 7E–G). As expected, ectopic overexpression of TRIM65 had no influence on the G2/M phase arrest caused by BTG3 K41R (Fig. 7H). These results further confirmed that TRIM65 functions as an oncoprotein through K41 ubiquitination of BTG3.

### TRIM65 and BTG3 expression levels correlated with the clinical pathogenesis of RCC

**Fig. 8:**
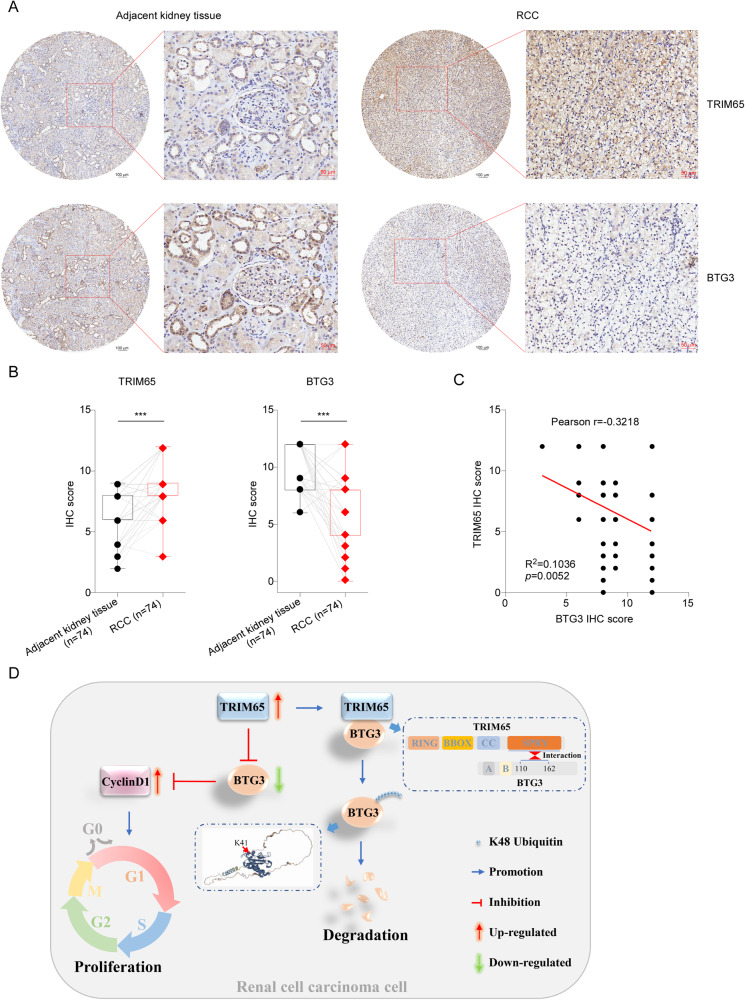
TRIM65 negatively correlates with BTG3 in clinical samples. **A** Representative IHC staining of TRIM65 and BTG3 on ccRCC and adjacent tissues. **B** IHC score of TRIM65 and BTG3. **C** The correlation of TRIM65 and BTG3 in clinical ccRCC samples. **D** Working model of TRIM65-BTG3-CyclinD1 axis in the proliferation of RCC cells.

At last, we evaluated the expression of TRIM65 and BTG3 protein and performed the correlation analysis in clinical samples provided by Shanghai Outdo Biotech. As shown in Fig. 8A, the representative images showed that the expression of TRIM65 in ccRCC tumor tissues was much higher while the BTG3 protein levels were lower than the adjacent normal kidney tissues, which was further confirmed by the results of the IHC scores (Fig. 8B). The results revealed a negative correlation between the protein levels of TRIM65 and BTG3 (Fig. 8C). The collective results showed that TRIM65 functioned as an oncogene in RCC via degradation of BTG3 (Fig. 8D).

## Discussion

Being a known E3 ligase, TRIM65 was involved in the regulation of some cell activities and related diseases mainly via the degradation of its specific substrates. As reported, TRIM65 functioned as an oncogene in various tumors, including breast cancer [32], gastric cancer [24], cervical cancer [23], pituitary tumors [33], etc. No study to date has mentioned the effects of TRIM65 on RCC. Thus, we first checked the expression of TRIM65 in clinical ccRCC tumors and adjacent normal kidney tissues through online databases. Results showed that TRIM65 was upregulated in ccRCC samples and higher TRIM65 levels were related to poor outcome in patients, which is consistent with the previous studies on other solid tumors.

In this study, we used cell counting/CCK8, colony formation, soft agar assay, and tumor xenografts to assess the effect of TRIM65 on the proliferation of RCC cells both in vitro and in vivo. Our findings proved that downregulation of TRIM65 can markedly suppress renal cancer cell proliferation as well as anchorage-independent growth, which was consistent with TRIM65 overexpression data. Results of soft agar assay indicated that knockdown of TRIM65 can not only decrease the number of cell colonies but also reduce the average area of colonies. Since the anchorage-independent growth of cancer cells is a key aspect of tumor phenotype which was related to the metastatic potential [34], we then explored the role of TRIM65 in the migration and invasion of RCC cells as well. We observed that downregulation of TRIM65 can also significantly suppress the migration and invasion of tumor cells (data not shown), the mechanism and detailed phenotype will be further studied.

To explore the underlying mechanism, we performed a classical yeast-two-hybrid assay to screen the binding partners of TRIM65. Based on our previous research experience [35, 36], the yeast-two-hybrid system used here was reliable with reduced false positive rate in protein-protein interaction. After sequencing and alignment, part of the candidate genes selected via the yeast-two-hybrid system which were relevant to tumor occurrence and progression were listed in Table S3. The number of positive colonies of BTG3 was 3, which is the highest among all these genes. Besides, BTG3 was known as a tumor suppressor in many tumors, which is critical in cell proliferation. Thus, BTG3 was selected as the critical candidate for further investigation.

IF staining of ectopic TRIM65 in RCC cells showed that TRIM65 protein is located in the cytoplasm as punctual structures. Consistently, Li et al. reported that endogenous TRIM65 can be found in punctate cytosolic spots in Hela cells [17], which assembles P-body structures linked to post-transcriptional regulation [37]. To explore the detailed location of these TRIM65 punctual spots, we co-stained TRIM65 and markers of some subcellular organelles (ER: Calnexin; Golgi: GolGA2/GM130; lysosome: CD107b/LAMP2; endosome: EEA1). Results revealed that TRIM65 can partially colocalize with EEA1 and LAMP2, but not with other proteins (Fig. S7). The reason why these spots formed and their detailed function remains unclear, which needs further studies.

Here, we provided evidence showing that BTG3 protein levels were negatively correlated with the protein levels of TRIM65, and BTG3 can be degraded through the ubiquitin-proteasome pathway. Further results showed that TRIM65 promoted the K48-linked but not K63-linked ubiquitination of BTG3, which led to its degradation. The ubiquitination site in BTG3 was also determined to be K41 using site-mutation strategies. Our findings provide a novel mechanism for the modulation of BTG3 protein levels and its function in tumor cells and the TRIM65-BTG3 interaction may play a key role in controlling the RCC cell proliferation.

Since we’ve proved that TRIM65 can directly bind to BTG3 and facilitate its degradation via K48-linked ubiquitination, then we designed and performed rescue assays to confirm the role of TRIM65-regulated BTG3 degradation on the proliferation of tumor cells as well as the cell cycle. As expected, upregulation of BTG3 can significantly suppress the oncogenic effects of TRIM65 on RCC cell growth and cell cycle. Consistently, downregulation of BTG3 via RNAi successfully abolished the CyclinD1 decline induced by TRIM65 knockdown (Fig. S6B). Moreover, TRIM65 overexpression can only regulate the anti-tumor effect of BTG3 rather than BTG3 K41R, indicating that TRIM65-regulated BTG3 ubiquitination and degradation are critical for its function in RCC.

It has been reported that the knockdown of BTG3 in cancer cells can lead to an accelerated exit from DNA damage-induced G2/M block [28]. Thus, we then detected the effect of TRIM65-mediated BTG3 degradation on the cell cycle. We first detected the cell cycle through flow cytometry, after confirming that TRIM65 has a regulatory effect on the renal cancer cell cycle, we also conducted a preliminary exploration of the existing key factors in our research group. Our results showed that TRIM65 relieved G2/M phase cell cycle arrest and increased the protein levels of CyclinD1, indicating that TRIM65 regulates the RCC cell cycle through BTG3-CyclinD1 axis. To clarify the role of CyclinD1 in the oncogenic functions of TRIM65, we inhibited CyclinD1 via CDK4/CyclinD1 inhibitor Fascaplysin and detected cell proliferation. After Fascaplysin treatment, overexpression of TRIM65 cannot promote cell growth (Fig. S8), indicating that TRIM65 functioned as an oncogene dependent on CyclinD1.

CyclinD1 is an important positive regulator of the cell cycle, it plays an important role in the occurrence and development of various tumors. In tumor tissue, the amplification or expression level of this gene is increased, which can promote tumor cell proliferation, DNA damage repair, invasion, and metastasis, and is an important proto-oncogene [38, 39]. Recently, Liu et al. discovered that ASF1B functioned as a proto-oncogene to modulate the G2/M phase and regulate the expression of cyclinD1, thereby affecting the occurrence and development of tumors [40], which is consistent with the function of TRIM65 that we explored in renal cancer. As the high expression is detected in more and more cancers, CyclinD1 has shown positive clinical value in the early classification, diagnosis, treatment, and prognosis prediction of various tumors [41, 42]. Based on qPCR and western blotting results (data not shown), we found that TRIM65 can only regulate the mRNA and protein levels of cyclinD1 consistently, but did not affect the expression of p27 and p21. Thus, we reached a preliminary conclusion that TRIM65 may promote RCC cell proliferation via the BTG3-cyclinD1 axis. However, we cannot exclude other target genes, which may be involved in the function of TRIM65. Next, we will continue to study the correlation between BTG3 and related transcription factors or target genes to search for other key downstream proteins.

In summary, we identified TRIM65 as a novel oncogene in RCC, which can promote cell proliferation both in vitro and in vivo via regulating BTG3 protein ubiquitination and degradation. TRIM65-mediated BTG3 degradation may be a potential mechanism that controls cell cycle progression and cell proliferation. Targeting the TRIM65/BTG3/ CyclinD1 axis may serve as a useful strategy for RCC treatment.

## Materials and methods

### Cell lines

Human renal cell carcinoma cell lines (ACHN, 769-P, Caki-1, Caki-2) were purchased from Procell Life Science&Technology (Wuhan, China) between 2019 and 2020, renal cell HK-2 and HEK293T were gifts from Dr. Chao Shen and Dr. Congyi Zheng (College of Life Sciences, Wuhan University) in March 2019. ACHN, 769-P, Caki-1, Caki-2, HK-2, and 293T cells were authenticated using short tandem repeat (STR) profiling in December 2019 and were negative for mycoplasma contamination detecting via PCR-based assay in October 2023. Cells were maintained routinely in RPMI 1640 medium, MEM, DMEM, McCoy’s 5 A (Procell, Wuhan, China) supplemented with 10% FBS (ExCell Bio, Taicang, China) and 100 units/ml penicillin and streptomycin under an atmosphere of 5% CO_2_ at 37 °C.

### Reagents

MG132, CHX, TRIM65 antibody (HPA021578) was purchased from Sigma-Aldrich (St.Louis, MO, USA). Fascaplysin was purchased from GlpBio (Montclair, CA, USA). BTG3 antibody (TA323849) was from OriGene (Rockville, MD, USA). K63-linkage specific Ub (5621 S), K48-linkage specific Ub (8081 S), and Ub (3936 S) were purchased from Cell Signaling Technology (Danvers, MA, USA). GFP (66002-1-Ig), FLAG (66008-4-Ig, 20543-1-Ig), HA (66006-2-Ig, 51064-2-Ig), Myc (60003-2-Ig), CyclinD1 (60186-1-Ig), Ki67 (27309-1-AP), PCNA (10205-2-AP), Calnexin (66903-1-Ig), GolGA2/GM130 (66662-1-Ig), CD107b/LAMP2 (66301-1-Ig), EEA1(68065-1-Ig), GAPDH (60004-1-Ig), β-actin (66009-1-Ig) were from Proteintech (Wuhan, China). Q-PCR primers were from TSINGKE Biological Technology (Beijing, China). BTG3 siRNA were purchased from OBIO (Shanghai, China), the siRNA sequence was: 5′-GGACAGGCCUACAGAUGUAUUTT-3′.

### Vectors and lentivirus infection

TRIM65 shRNA vectors (the miRNAi vectors) were generated by the BLOCK-iT Pol II miR-RNAi Expression kit (Invitrogen, Waltham, MA, USA), and then subcloned to pLVX-puro-IRES-GFP-shRNA vector. The miRNAi target sequences were: shRNA-1: 5′- TCCTCAGTGGACAAACTGTGC -3′; shRNA-2: 5′- TATTTCCAGGGCCTGTA GCAG -3′.

The lentiviruses used for overexpression or knockdown of TRIM65 were generated, packaged, and purified according to the methods described before [43]. Stable cell lines overexpressing or knockdown of TRIM65 were generated by lentivirus transduction and antibiotic selection. Simply, the lentiviruses carrying the target gene or shRNA were produced by co-transfection of lentiviral transfer (pLVX-puro-IRES-GFP), and packaging plasmids pMD2G and psPAX2 into HEK293T cells using the calcium phosphate transfection method. The lentivirus-containing supernatant was collected at 24 and 48 h after transfection. Cells were transduced by the lentivirus after 16 h in the presence of 8 μg/mL polybrene. To select for stable expression, cells were maintained in the growth medium with 2 μg/mL puromycin for 10 days. The selected stable cell clones were verified by western blotting and were used in various experiments.

### Cell proliferation assay

Cell counting kit-8 (CCK8), low serum, and saturation density assay were used to detect cell proliferation. For CCK8 (Dojindo, Kumamoto, Japan), cells were seeded at a density of 5000 cells in 96-well plates, and on the next day, 10 μL CCK8 solution was added to each well. The plates were incubated for 1 h at 37 °C and the absorbance of OD450 was measured immediately on the microplate reader. In the low serum assay, the indicated cells were inoculated in 12-well plates (10^5^ cells per well) and cultured overnight in full growth medium containing 10% FBS. On the second day, the number of cells was counted and recorded as the data of day 0, and the medium was changed to 1% FBS medium every other day for 6 consecutive days. The cells were counted with a hemocytometer at day 2, 4, and 6, respectively. For saturation density assay, cells were seeded in a 12-well plate at a density of 10^5^ cells with 10% FBS medium, with the medium being changed every other day. Cell density was determined by cell count at day 6.

### Colony formation and soft agar assay

For colony formation assay, 1000 cells were plated into 6-well plates and allowed to grow in 10% FBS medium for 12–14 days. Then, the plates were fixed and stained with 2% crystal violet. In the soft agar assay, lentivirus-infected stable cells were seeded in 6-well plates (10^4^ cells per well) with an upper layer of 0.35% agar growth medium containing cell suspension and a lower layer of 0.7% agar growth medium. The culture medium was replenished every 3 days, and the cells were cultured for about 10–20 days. Colonies ≥50 μm in diameter were imaged and counted, and the area of each colony was also calculated.

### Western blotting and quantitative real-time PCR (qPCR)

Whole-cell protein lysates were isolated and obtained by lysis buffer and after BCA quantitation via BCA Protein Assay Kit (Thermo Fisher, Waltham, MA, USA), protein extracts were analyzed through western blotting as described previously [36]. The density of the western blotting band was analyzed with Gelpro 32 software. qPCR was performed on a ViiA-7 real-time PCR system instrument (ABI, Waltham, MA, USA) using the 2^−ΔΔCT^ method compared with β-actin transcript, the primers’ sequences used were listed in Table S1.

### Tumor xenografts

To establish a tumor xenograft model, 5 × 10^6^ stably infected ACHN cells resuspended in 50 μL PBS (control group and TRIM65 group) were subcutaneously injected into both flanks of 5-week-old male Balb/c-nu mice (Hunan SJA Laboratory Animal Co., China). For TRIM65 knockdown experiments, 5 × 10^6^ stably infected 769-P cells/50 μL PBS (control group and TRIM65 shRNA-2 group) were collected and subcutaneously injected into 5-week-old male Balb/c-nu mice in the indicated positions. Tumor sizes were measured with calipers every 3 or 4 days, and the volumes of tumors were calculated via the formula (V = L × W^2^/2). On the day of sacrifice, the xenografts were weighed and harvested for IHC analysis.

### Yeast-two-hybrid

The yeast-two-hybrid screening was conducted using the Matchmaker Gold Yeast-Two-Hybrid System (Clontech, Beijing, China) according to the instructions of the manufacturer. Full-length cDNA of the human *TRIM65* gene was subcloned into the pGBKT7 vector as the bait. The Y2HGold yeasts which were transformed by pGBKT7-TRIM65 were then mated with the Y187 yeast strain library (Universal Human Mate & Plate Library, Clontech) at 30 °C with slow shaking for 20–24 h to form zygotes. After two rounds of screening, blue colonies on higher stringency agar plates (QDO/X/A: SD/-Ade/-His/-Leu/-Trp/X-α-Gal) were considered as positive candidates, which can be identified via PCR, sequencing, and nucleotide blast.

### Co-immunoprecipitation (Co-IP)

For co-IP, cells transfected with indicated vectors were harvested and solubilized in lysis buffer (0.5% Lubrol-Px, 50 mM KCl, 2 mM CaCl_2_, 20% glycerol, and 50 mM Tris-HCl, pH7.4). Cell lysates were incubated with 1 μg indicated antibodies for over 4 h at 4 °C, and then incubated with protein-A or G beads (Roche, Basilea, Switzerland) at 4 °C overnight. After being washed for 3 times, bound proteins were subjected to immunoblotting analysis as described previously [44].

### GST pull-down

To determine the direct interaction between TRIM65 and BTG3, recombinant GST-TRIM65 protein was produced and purified in *E*.*coli* as we’ve done before [29]. Cell lysates from HA-BTG3-transfected HEK293T cells were incubated with GST-TRIM65 or GST as negative control overnight with gentle rolling at 4 °C. Protein complexes were precipitated with Glutathione Sepharose 4B Beads (GE Healthcare, Chicago, IL, USA) and then used to SDS-PAGE (Coomassie brilliant blue staining) and immunoblotting with indicated antibodies.

### Immunofluorescence

To detect the co-localization of TRIM65 and BTG3, ACHN cells were plated on the coverslips and co-transfected with FLAG-TRIM65/HA-BTG3 for 24 h, then treated with or without MG132 for another 4 h. Cells were fixed in cold 4% PFA for over 1 h and incubated with 0.1% TritonX-100 at 4 °C for 30 min. For IF staining of TRIM65 (FLAG) and BTG3 (HA), the detailed methods described before were used [44]. After adding a drop of mounting medium, the slides were photographed using a LSM800 confocal microscope from Zeiss Company.

### Immunohistochemistry

Human RCC tissue microarray analysis was purchased from Shanghai Outdo Biotech (Shanghai, China). The IHC staining of TRIM65 and BTG3 was carried out using antibodies listed above with the detailed procedures described previously [45]. The tissue microarray images were then scanned and analyzed via Image Scope. IHC staining (Ki67, TRIM65, BTG3) of tumors from in vivo xenograft assay was performed as previously described [46]. The detailed pathological and clinical information for patients is shown in Table S2.

### Statistical analysis

Data were expressed as mean ± SEM from experiments based on at least three replicates. The two-tailed unpaired Student’s *t*-test was used to assess the differences between the two groups. *P*-value less than 0.05 was considered significant.

### Supplementary information

Supplementary figures and tables

Uncropped WB files

## Data Availability

All datasets generated for this study are included in the article/Supplementary Material.

## References

[1] Bray F, Ferlay J, Soerjomataram I, Siegel RL, Torre LA, Jemal A (2018). Global cancer statistics 2018: GLOBOCAN estimates of incidence and mortality worldwide for 36 cancers in 185 countries. CA Cancer J Clin.

[2] Padala SA, Barsouk A, Thandra KC, Saginala K, Mohammed A, Vakiti A (2020). Epidemiology of renal cell carcinoma. World J Oncol.

[3] National Health Commission of the People’s Republic of China. Chinese guidelines for diagnosis and treatment of renal cell carcinoma 2018 (English version). Chin J Cancer Res. 2019;31:29–48. 10.21147/j.issn.1000-9604.2019.02.01 PMC6513746 31156297

[4] Linehan WM, Ricketts CJ (2019). The Cancer Genome Atlas of renal cell carcinoma: findings and clinical implications. Nat Rev Urol.

[5] Hsieh JJ, Purdue MP, Signoretti S, Swanton C, Albiges L, Schmidinger M (2017). Renal cell carcinoma. Nat Rev Dis Primers.

[6] Ravaud A, Motzer RJ, Pandha HS, George DJ, Pantuck AJ, Patel A (2016). Adjuvant sunitinib in high-risk renal-cell carcinoma after nephrectomy. N Engl J Med.

[7] Weissman AM (1997). Regulating protein degradation by ubiquitination. Immunol Today.

[8] Park J, Cho J, Song EJ (2020). Ubiquitin-proteasome system (UPS) as a target for anticancer treatment. Arch Pharm Res.

[9] Cornelius RJ, Ferdaus MZ, Nelson JW, McCormick JA (2019). Cullin-Ring ubiquitin ligases in kidney health and disease. Curr Opin Nephrol Hypertens.

[10] Zhang E, Chen S, Tang H, Fei C, Yuan Z, Mu X (2022). CDK1/FBXW7 facilitates degradation and ubiquitination of MLST8 to inhibit progression of renal cell carcinoma. Cancer Sci.

[11] Dong H, Zhu L, Sun J, Zhang Y, Cui Q, Wu L (2021). Pan-cancer analysis of NEDD4L and its tumor suppressor effects in clear cell renal cell carcinoma. J Cancer.

[12] Miao C, Liang C, Li P, Liu B, Qin C, Yuan H (2021). TRIM37 orchestrates renal cell carcinoma progression via histone H2A ubiquitination-dependent manner. J Exp Clin Cancer Res.

[13] Xiao C, Zhang W, Hua M, Chen H, Yang B, Wang Y (2021). TRIM27 interacts with Ikappabalpha to promote the growth of human renal cancer cells through regulating the NF-kappaB pathway. BMC Cancer.

[14] Lu Y, Qin H, Jiang B, Lu W, Hao J, Cao W (2021). KLF2 inhibits cancer cell migration and invasion by regulating ferroptosis through GPX4 in clear cell renal cell carcinoma. Cancer Lett.

[15] Yamada Y, Kimura N, Takayama KI, Sato Y, Suzuki T, Azuma K (2020). TRIM44 promotes cell proliferation and migration by inhibiting FRK in renal cell carcinoma. Cancer Sci.

[16] Fornage M, Debette S, Bis JC, Schmidt H, Ikram MA, Dufouil C (2011). Genome-wide association studies of cerebral white matter lesion burden: the CHARGE consortium. Ann Neurol.

[17] Li S, Wang L, Fu B, Berman MA, Diallo A, Dorf ME (2014). TRIM65 regulates microRNA activity by ubiquitination of TNRC6. Proc Natl Acad Sci USA.

[18] Lang X, Tang T, Jin T, Ding C, Zhou R, Jiang W (2017). TRIM65-catalized ubiquitination is essential for MDA5-mediated antiviral innate immunity. J Exp Med.

[19] Meng J, Yao Z, He Y, Zhang R, Zhang Y, Yao X (2017). ARRDC4 regulates enterovirus 71-induced innate immune response by promoting K63 polyubiquitination of MDA5 through TRIM65. Cell Death Dis.

[20] Chen D, Li Y, Zhang X, Wu H, Wang Q, Cai J (2019). Ubiquitin ligase TRIM65 promotes colorectal cancer metastasis by targeting ARHGAP35 for protein degradation. Oncogene.

[21] Wei WS, Chen X, Guo LY, Li XD, Deng MH, Yuan GJ (2018). TRIM65 supports bladder urothelial carcinoma cell aggressiveness by promoting ANXA2 ubiquitination and degradation. Cancer Lett.

[22] Yang YF, Zhang MF, Tian QH, Zhang CZ (2017). TRIM65 triggers beta-catenin signaling via ubiquitylation of Axin1 to promote hepatocellular carcinoma. J Cell Sci.

[23] Wang XY, Mao HW, Guan XH, Huang QM, Yu ZP, Wu J (2022). TRIM65 promotes cervical cancer through selectively degrading p53-mediated inhibition of autophagy and apoptosis. Front Oncol.

[24] Liu C, Sun W, Yang K, Xia B (2022). Knockdown of TRIM65 suppressed the proliferation and invasiveness of gastric cancer cells by restricting the ubiquitin degradation of PPM1A. Exp Cell Res.

[25] Cheng YC, Lin TY, Shieh SY (2013). Candidate tumor suppressor BTG3 maintains genomic stability by promoting Lys63-linked ubiquitination and activation of the checkpoint kinase CHK1. Proc Natl Acad Sci USA.

[26] Mao D, Qiao L, Lu H, Feng Y (2016). B-cell translocation gene 3 overexpression inhibits proliferation and invasion of colorectal cancer SW480 cells via Wnt/beta-catenin signaling pathway. Neoplasma.

[27] An Q, Zhou Y, Han C, Zhou Y, Li F, Li D (2017). BTG3 overexpression suppresses the proliferation and invasion in epithelial ovarian cancer cell by regulating AKT/GSK3beta/beta-catenin signaling. Reprod Sci.

[28] Ou YH, Chung PH, Hsu FF, Sun TP, Chang WY, Shieh SY (2007). The candidate tumor suppressor BTG3 is a transcriptional target of p53 that inhibits E2F1. EMBO J.

[29] Li Y, Huang X, Guo F, Lei T, Li S, Monaghan-Nichols P (2020). TRIM65 E3 ligase targets VCAM-1 degradation to limit LPS-induced lung inflammation. J Mol Cell Biol.

[30] Huang Y, Xiao Y, Zhang X, Huang X, Li Y (2021). The emerging roles of tripartite motif proteins (TRIMs) in acute lung injury. J Immunol Res.

[31] Lv C, Wang H, Tong Y, Yin H, Wang D, Yan Z (2018). The function of BTG3 in colorectal cancer cells and its possible signaling pathway. J Cancer Res Clin Oncol.

[32] Lu Y, Xiao Y, Yang J, Su H, Zhang X, Su F (2022). TRIM65 promotes malignant cell behaviors in triple-negative breast cancer by impairing the stability of LATS1 protein. Oxid Med Cell Longev.

[33] Yao H, Xie W, Dai Y, Liu Y, Gu W, Li J (2022). TRIM65 determines the fate of a novel subtype of pituitary neuroendocrine tumors via ubiquitination and degradation of TPIT. Neuro Oncol.

[34] Mori S, Chang JT, Andrechek ER, Matsumura N, Baba T, Yao G (2009). Anchorage-independent cell growth signature identifies tumors with metastatic potential. Oncogene.

[35] Wang Y, Li Y, Hu G, Huang X, Rao H, Xiong X (2016). Nek2A phosphorylates and stabilizes SuFu: a new strategy of Gli2/Hedgehog signaling regulatory mechanism. Cell Signal.

[36] Li Y, Zhang Q, Li N, Ding L, Yi J, Xiao Y (2021). Ataxin-10 inhibits TNF-alpha-induced endothelial inflammation via suppressing interferon regulatory factor-1. Mediators Inflamm.

[37] Luo Y, Na ZK, Slavoff SA (2018). P-bodies: composition, properties, and functions. Biochemistry.

[38] John RR, Malathi N, Ravindran C, Anandan S (2017). Mini review: multifaceted role played by cyclin D1 in tumor behavior. Indian J Dent Res.

[39] Yu Z, Wang L, Wang C, Ju X, Wang M, Chen K (2013). Cyclin D1 induction of Dicer governs microRNA processing and expression in breast cancer. Nat Commun.

[40] Liu X, Song J, Zhang Y, Wang H, Sun H, Feng X (2020). ASF1B promotes cervical cancer progression through stabilization of CDK9. Cell Death Dis.

[41] Li G, Yang T, Chen Y, Bao J, Wu D, Hu X (2021). USP5 sustains the proliferation of glioblastoma through stabilization of cyclinD1. Front Pharmacol.

[42] Hao J, Zhang W, Lyu Y, Zou J, Zhang Y, Lyu J (2021). Combined use of cyclinD1 and Ki67 for prognosis of luminal-like breast cancer patients. Front Oncol.

[43] Deng Y, Peng D, Xiao J, Zhao Y, Ding W, Yuan S (2023). Inhibition of the transcription factor ZNF281 by SUFU to suppress tumor cell migration. Cell Death Differ..

[44] Shao J, Xu L, Chen L, Lu Q, Xie X, Shi W (2017). Arl13b promotes gastric tumorigenesis by regulating smo trafficking and activation of the hedgehog signaling pathway. Cancer Res.

[45] Han T, Zhan W, Gan M, Liu F, Yu B, Chin YE (2018). Phosphorylation of glutaminase by PKCepsilon is essential for its enzymatic activity and critically contributes to tumorigenesis. Cell Res.

[46] Ding L, Fang Y, Li Y, Hu Q, Ai M, Deng K (2021). AIMP3 inhibits cell growth and metastasis of lung adenocarcinoma through activating a miR-96-5p-AIMP3-p53 axis. J Cell Mol Med.

