# Supplementary material for: TRIM65 promotes renal cell carcinoma through ubiquitination and degradation of BTG3
Source: Cell Death Dis. 2024 May 22;15(5):355. doi: 10.1038/s41419-024-06741-3 (PMC11111765; doi:10.1038/s41419-024-06741-3)
Supplement: Supplementary file 1 — Supplementary figures and tables [file 41419_2024_6741_MOESM1_ESM.pdf]

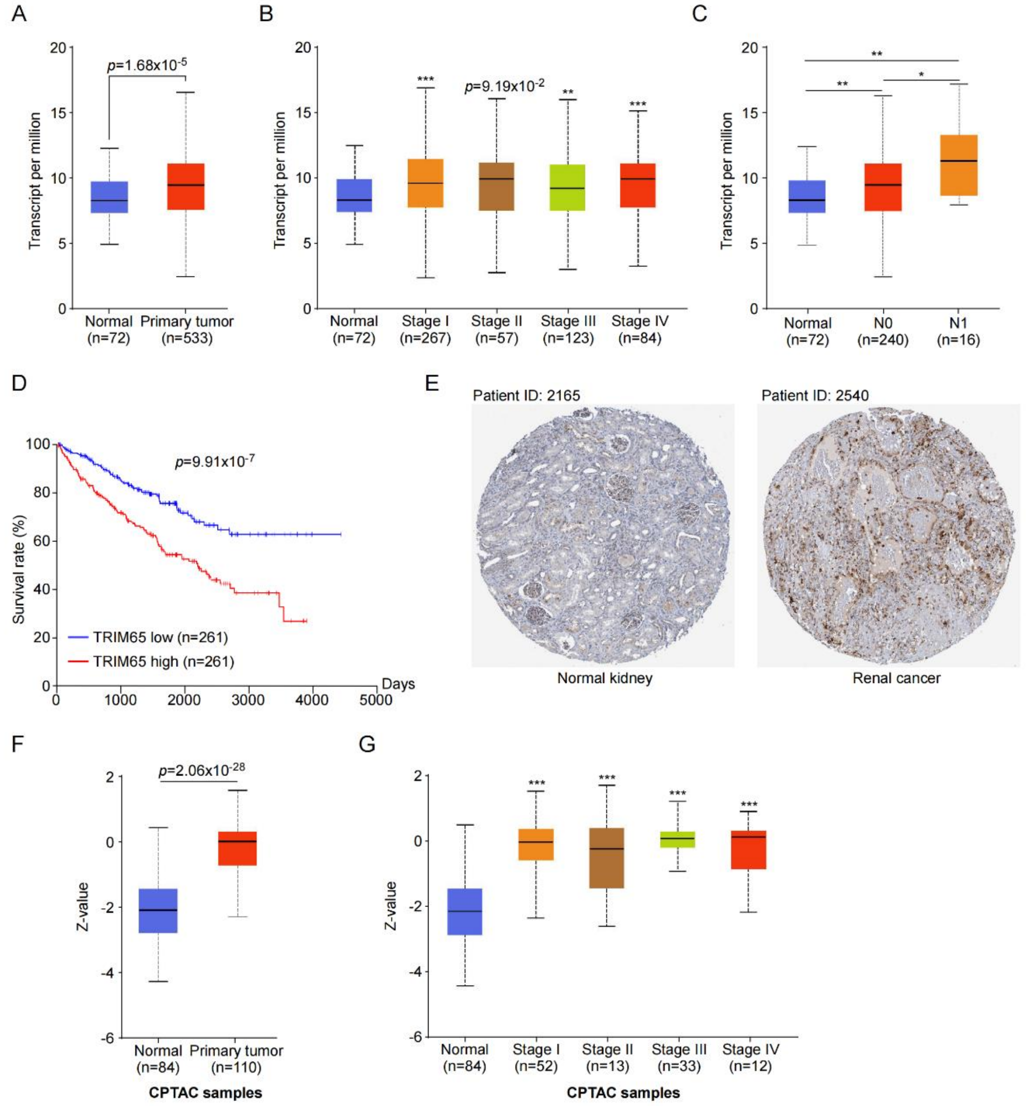


**Figure S1:TRIM65 is highly expressed in human renal cell carcinoma tissues**

**A.** The mRNA expression of TRIM65 in renal cell carcinoma was higher than that in normal renal tissues. **B-C**. The mRNA levels of TRIM65 in ccRCC were associated with different tumor stages (B) and lymph node metastasis status (C). **D**. The prognostic value of TRIM65 for overall survival was determined by Kaplan-Meier analyses. Data is from TCGA's renal cell carcinoma data set. **E.** The representative IHC staining images of TRIM65 from HPA on normal kidney and renal cancer. **F.** The protein levels of TRIM65 in ccRCC tumors and normal tissues. **G.** TRIM65 protein levels in normal kidneys and ccRCC tumors with different stages.

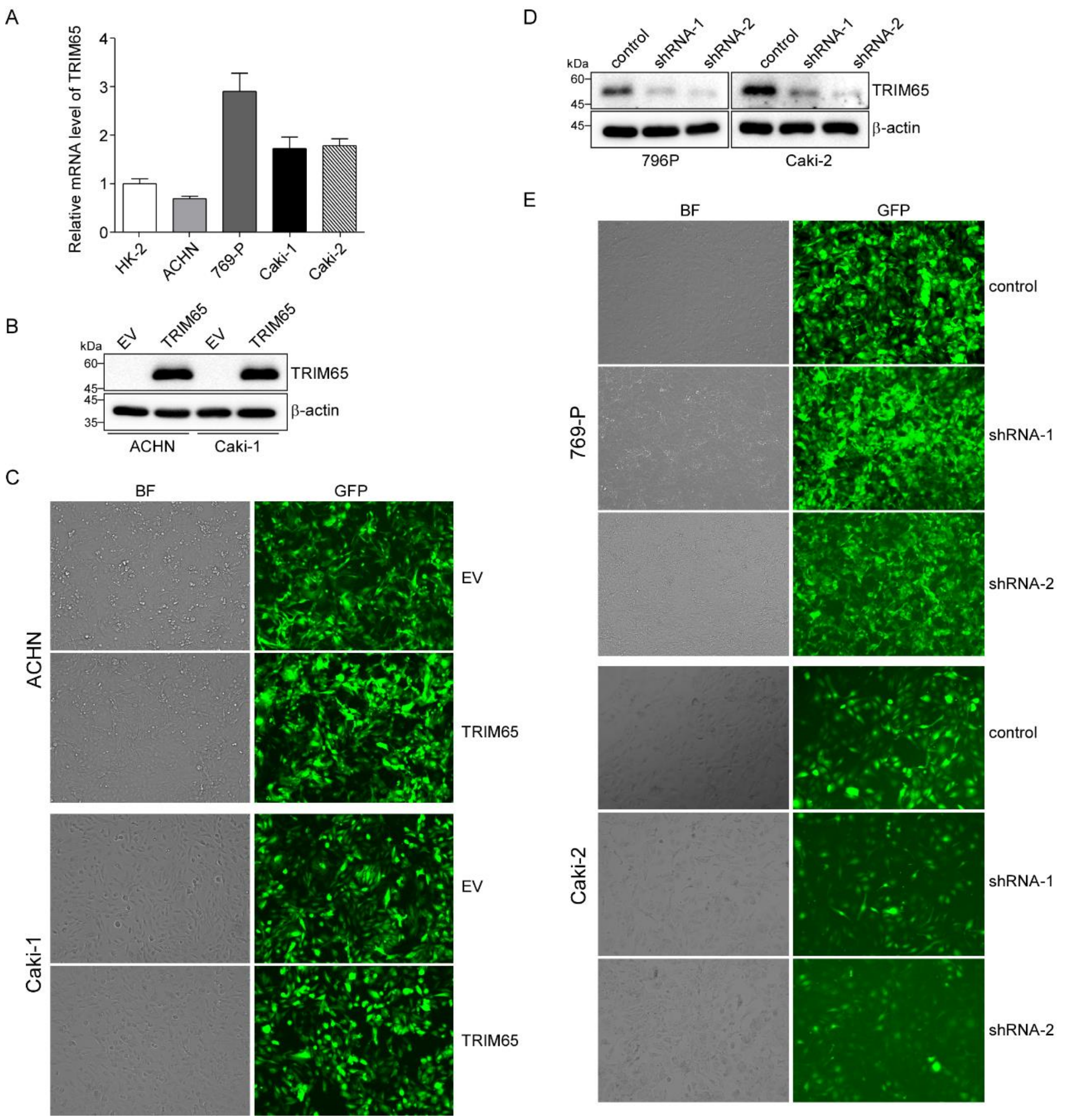


**Figure S2: Construction of stable cell lines overexpressing and knockdown of TRIM65 by lentivirus infection**

**A.** The mRNA levels of TRIM65 in the indicated cell lines were detected by real-time quantitative PCR. **B.** Western blotting analysis of TRIM65 overexpressed cells. **C.** Representative fluorescent images of TRIM65 overexpressed cells. **D.** Western blotting analysis of TRIM65 knockdown cells. **E.** Representative fluorescent images of TRIM65 knockdown cells.

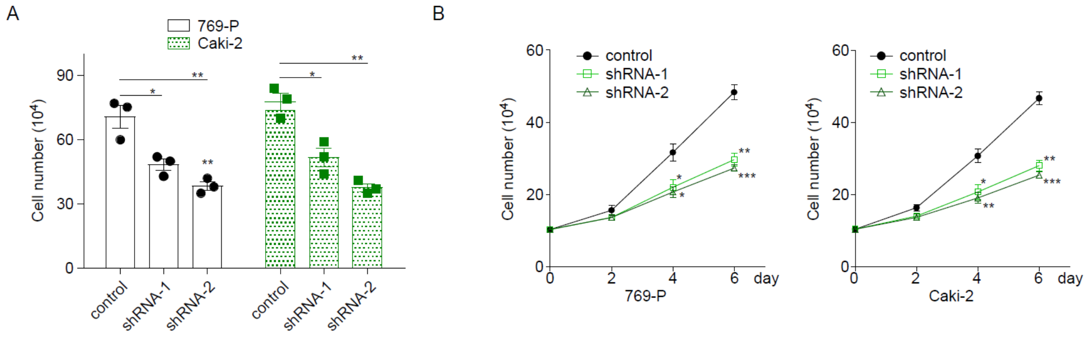


**Figure S3: Cell counting of TRIM65 knockdown cells**

**A**. Saturation density assay. TRIM65 knockdown and control stable cells were plated and cultured in full growth medium for 6 days, and then were trypsinized and counted. Data are presented as mean ± SEM (n = 3). **B.** Low serum assay. TRIM65 stably knockdown cells and control cells were cultured in 1% FBS medium. At the indicated times, cells were trypsinized and counted. $^*p < 0.05$, $^{**}p < 0.01$, $^{***}p < 0.001$ by Student's t test.

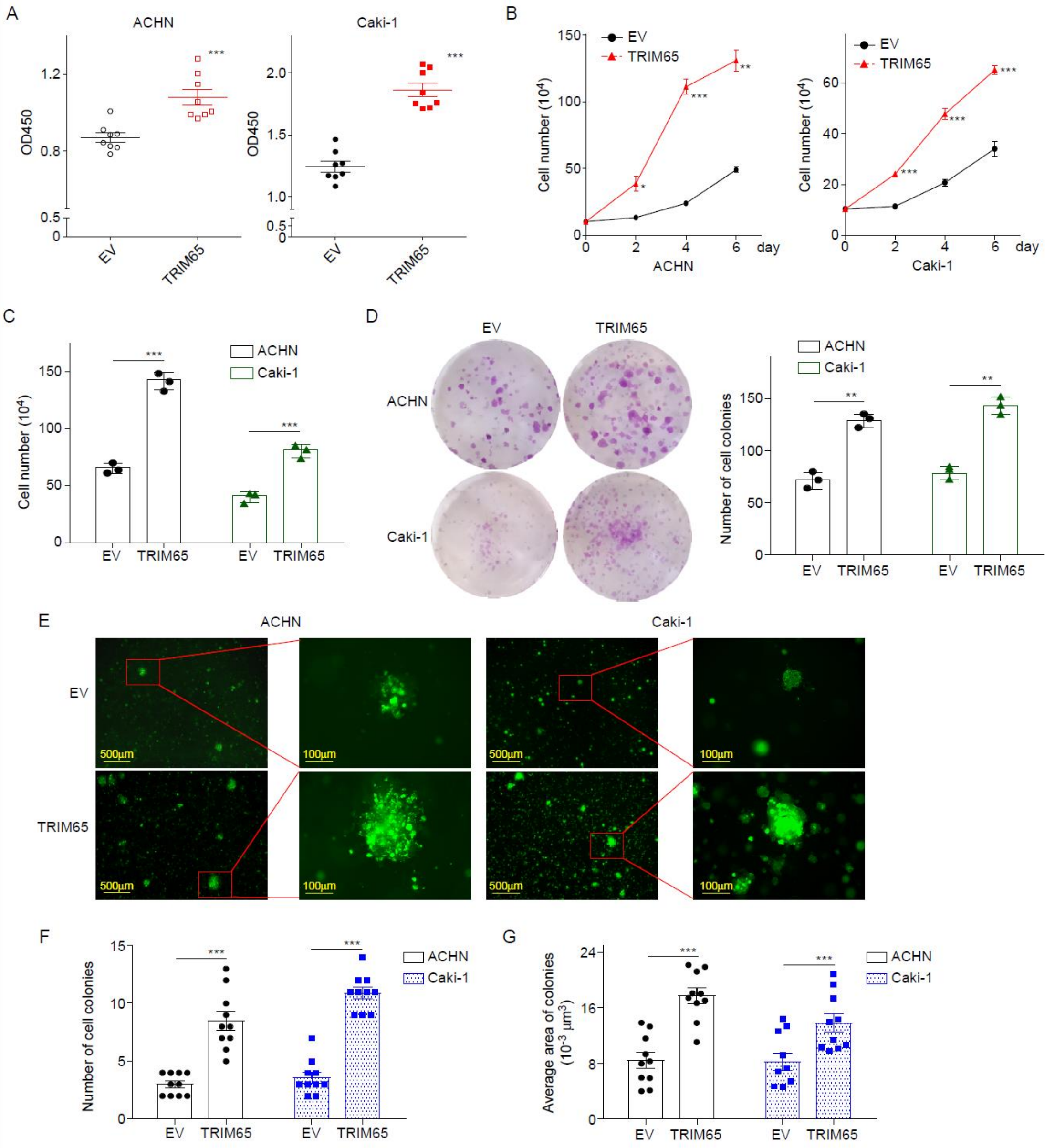


**Figure S4: TRIM65 overexpression promoted the proliferation of RCC cells**

**A.** CCK-8 assay. ACHN and Caki-1 cells were infected with lentivirus to construct stable cell lines overexpressing TRIM65 or empty vector (EV). **B.** Low serum assay. **C.** Saturation density assay. **D.** Colony formation assay. The number of colonies were counted and showed in the right panel. **E.** Soft agar colony formation assay. Colonies larger than 50 µm were imaged and counted. **F**. The number of cell colonies were calculated in ten random selected views. Data are presented as mean ± SEM (n = 10). **G.** The average area of colonies was calculated in ten random selected views. Data are presented as mean ± SEM (n = 10). **p* < 0.05, ***p* < 0.01, ****p* < 0.001 by Student's t test.

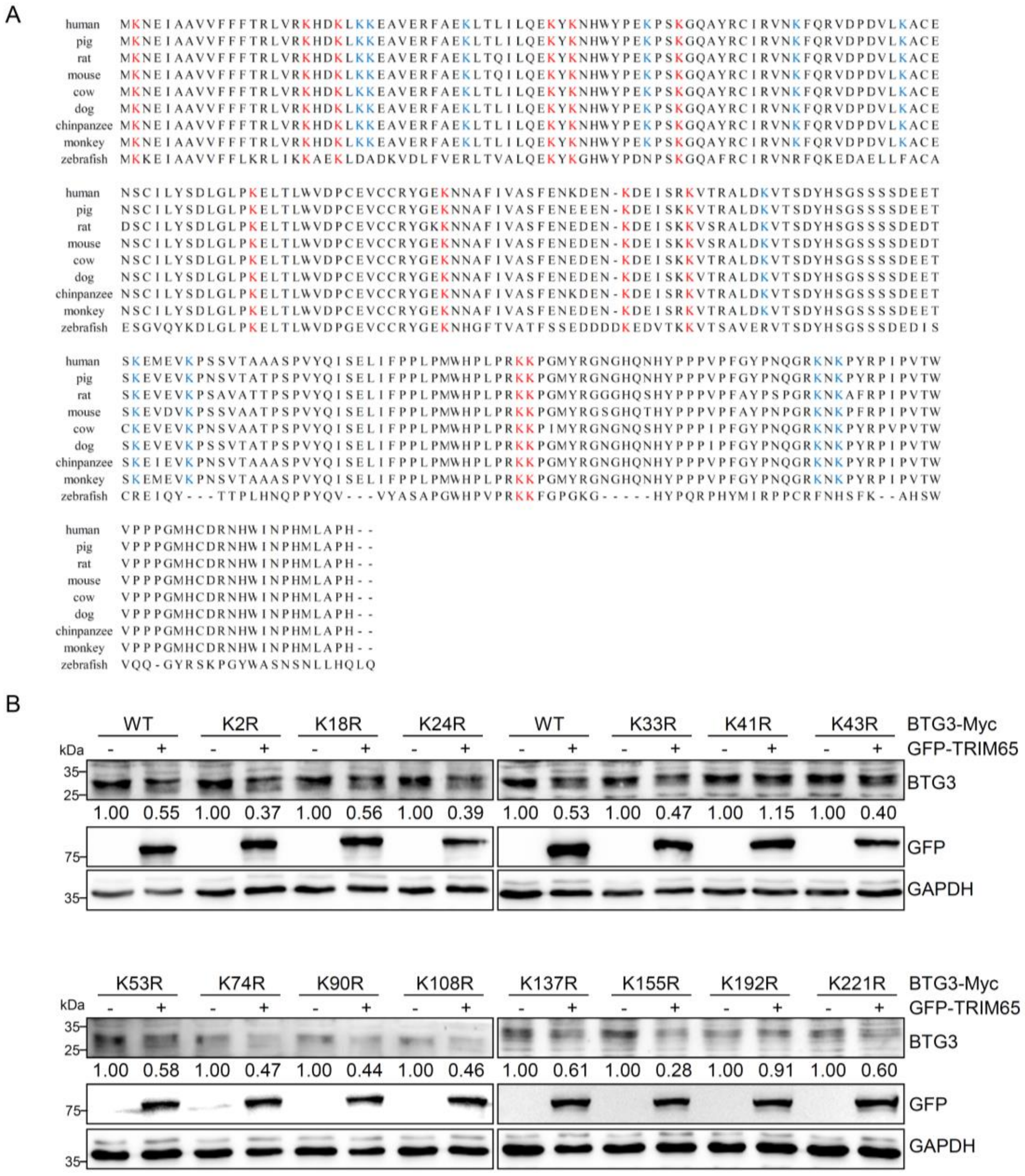

**Figure S5: Screening of the ubiquitination site of TRIM65 on BTG3**

**A**. Alignment of BTG3 protein sequences from different species. **B.** A series of BTG3 mutant vectors were constructed as indicated. HEK293T cells were co-transfected with GFP-TRIM65 and BTG-Myc or BTG-Myc mutants. The protein levels of BTG3 were then determined by immunoblotting.

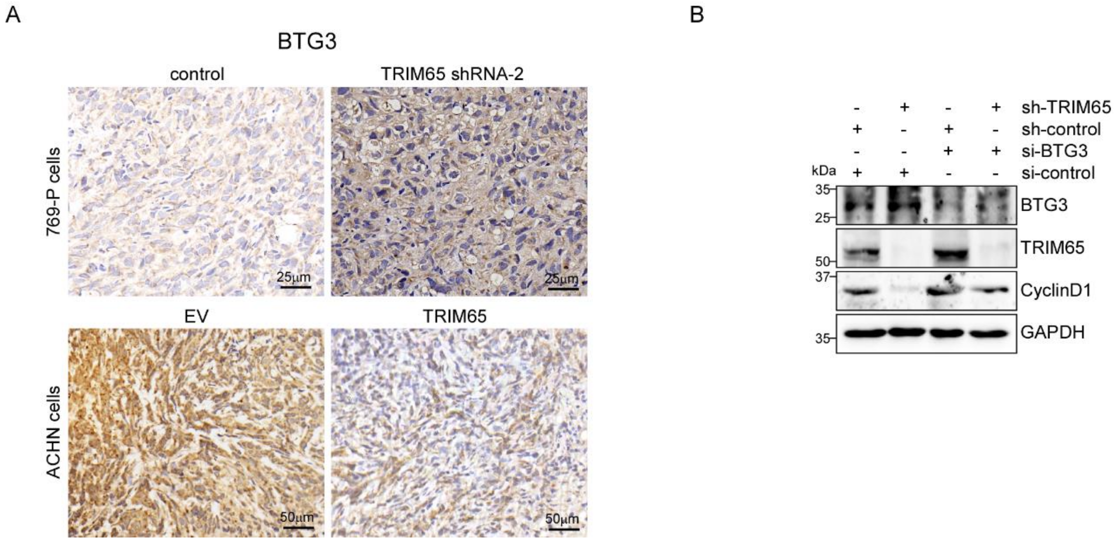


**Figure S6: TRIM65 regulates the expression of BTG3 and CyclinD1**
**A.** The IHC staining of BTG3 in tumors of 769-P and ACHN cells in xenograft model as indicated. **B.** Western blotting analysis of 4 groups for co-transfection into 769-P cells.

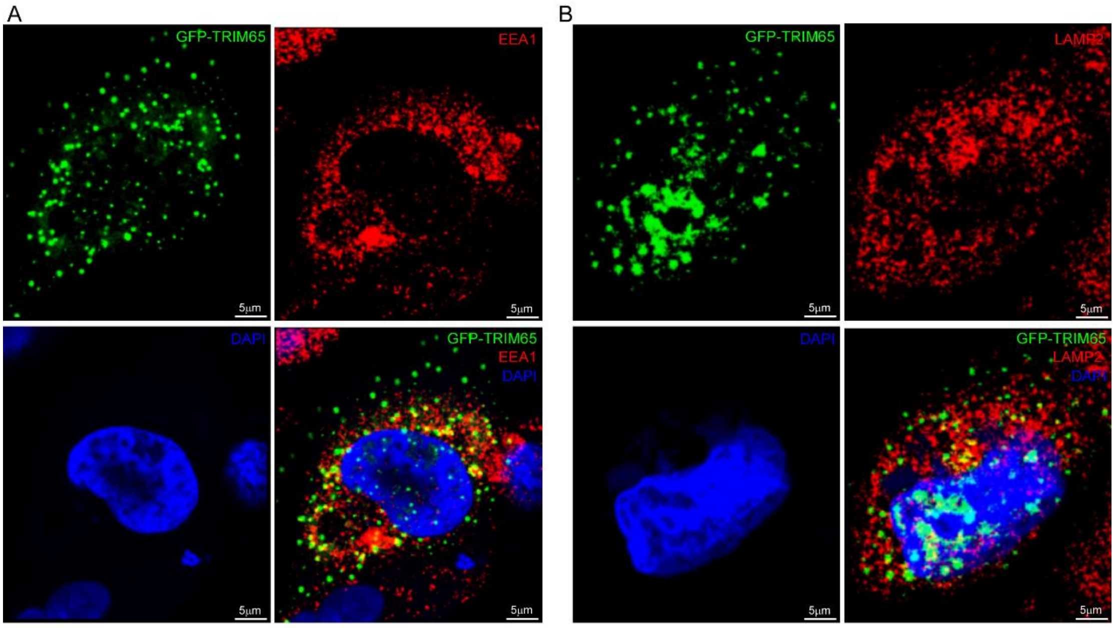


**Figure S7: Sub-cellular localization of TRIM65**
Co-localization of TRIM65 and organelles (endosome and lysosome) in RCC cells. ACHN cells were co-stained with antibodies against GFP or EEA1**(A)**/LAMP2**(B)**.

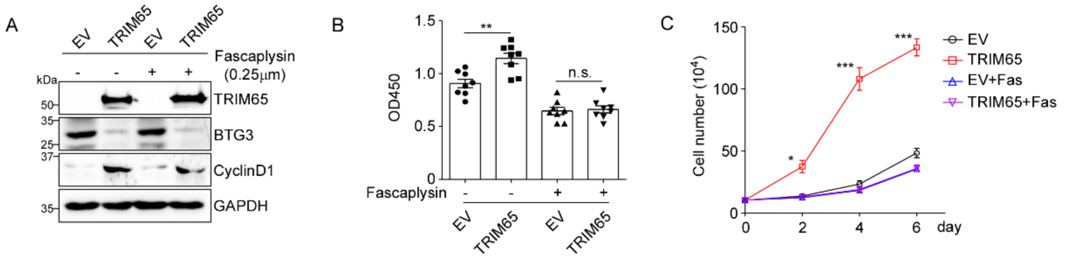


**Figure S8: Inhibition of CyclinD1 restrained the oncogenic effect of TRIM65 on cell growth**
**A.** Western blotting analysis of 4 groups of ACHN cells. Cells were treated with 0.25μM Fascaplysin for 24h. **B.** CCK-8 assay. **C.** Low serum assay.

Table S1 Sequence of primers used in qRT-PCR

| Primer name | Primer sequences |
|---|---|
| TRIM65-Forward primer | GCTCTGGCAGAATTATCGCAATC |
| TRIM65-Reverse primer | AAATGGTGTGTCACCCGCTG |
| β-actin- Forward primer | GATATCGCTGCGCTGGTCG |
| β-actin- Reverse primer | CATTCCCACCATCACACCCT |

Table S2

Association of TRIM65 and BTG3 expression levels with different clinicopathologic characteristics in ccRCC

| Clinicopathologic | | TRIM65 expression | | | | | BTG3 expression | | | | |
|---|---|---|---|---|---|---|---|---|---|---|---|
| | | low | | High | | P value | low | | High | | P value |
| Measurement data | n | Mean | SD | Mean | SD | | Mean | SD | Mean | SD | |
| Age(y) | 73 | 56.75 | 17.05 | 56.45 | 10.48 | 0.962 | 54.56 | 11.94 | 58.15 | 10.29 | 0.174 |
| Tumor size(cm3) | 73 | 111.82 | 104.43 | 221.99 | 255.07 | 0.035* | 200.07 | 225.38 | 218.5 | 263.7 | 0.749 |
| Enumeration data | n | Count | n% | Count | n% | P | Count | n% | Count | n% | P |
| **Gender** | | | | | | | | | | | |
| Male | | 3 | 4.11% | 47 | 64.38% | | 21 | 28.77% | 29 | 39.72% | |
| Female | | 5 | 6.85% | 18 | 24.66% | | 13 | 17.81% | 10 | 13.70% | |
| Total | 73 | 8 | 10.96% | 65 | 89.04% | 0.046* | 34 | 46.58% | 39 | 53.42% | 0.248 |
| **Pathological Grading** | | | | | | | | | | | |
| 1 | | 1 | 1.37% | 2 | 2.74% | | 2 | 2.74% | 1 | 1.37% | |
| 2 | | 5 | 6.85% | 49 | 67.12% | | 24 | 32.88% | 30 | 41.09% | |
| 3 | | 2 | 2.74% | 13 | 17.81% | | 8 | 10.96% | 7 | 9.59% | |
| 4 | | 0 | 0% | 1 | 1.37% | | 0 | 0% | 1 | 1.37% | |
| Total | 73 | 8 | 10.96% | 65 | 89.04% | 0.149 | 34 | 46.58% | 39 | 53.42% | 0.149 |
| **Distant invasion** | | | | | | | | | | | |
| YES | | 0 | 0% | 2 | 2.74% | | 0 | 0% | 2 | 2.74% | |
| NO | | 8 | 10.96% | 63 | 86.30% | | 34 | 46.58% | 37 | 50.68% | |
| Total | 73 | 8 | 10.96% | 65 | 89.04% | 0.615 | 34 | 46.58% | 39 | 53.42% | 0.181 |
| **Vascular invasion** | | | | | | | | | | | |
| YES | | 6 | 8.22% | 58 | 79.45% | | 31 | 42.47% | 35 | 47.94% | |
| NO | | 2 | 2.74% | 7 | 9.59% | | 3 | 4.11% | 4 | 5.48% | |
| Total | 73 | 8 | 10.96% | 65 | 89.04% | 0.248 | 34 | 46.58% | 39 | 53.42% | 0.836 |
| **T factor** | | | | | | | | | | | |

| | | | | | | | | | | | |
|---|---|---|---|---|---|---|---|---|---|---|---|
| T1 | | 4 | 5.48% | 32 | 43.83% | | 18 | 24.66% | 18 | 24.66% | |
| T2 | | 2 | 2.74% | 15 | 20.55% | | 8 | 10.96% | 9 | 12.32% | |
| T3 | | 2 | 2.74% | 14 | 19.18% | | 4 | 5.48% | 12 | 16.44% | |
| T4 | | 0 | 0% | 4 | 5.48% | | 4 | 5.48% | 0 | 0% | |
| Total | 73 | 8 | 10.96% | 65 | 89.04% | 0.031* | 34 | 46.58% | 39 | 53.42% | 0.031* |
| **M factor** | | | | | | | | | | | |
| M0 | | 8 | 10.96% | 63 | 86.30% | | 34 | 46.58% | 37 | 50.68% | |
| M1 | | 0 | 0 | 2 | 2.74% | | 0 | 0% | 2 | 2.74% | |
| Total | 73 | 8 | 10.96% | 65 | 89.04% | 0.744 | 34 | 46.58% | 39 | 53.42% | 0.744 |
| **Clinical stage** | | | | | | | | | | | |
| stage1 | | 4 | 5.48% | 31 | 42.47% | | 18 | 24.66% | 17 | 23.29% | |
| stage2 | | 2 | 2.74% | 15 | 20.55% | | 8 | 10.96% | 9 | 12.32% | |
| stage3 | | 2 | 2.74% | 13 | 17.81% | | 4 | 5.48% | 11 | 15.07% | |
| stage4 | | 0 | 0% | 6 | 8.21% | | 4 | 5.48% | 2 | 2.74% | |
| Total | 73 | 8 | 10.96% | 65 | 89.04% | 0.149 | 34 | 46.58% | 39 | 53.42% | 0.021* |

Table S3. Part of tumor-related genes selected via yeast-two hybrid system

| Name | Gene ID | Number of colonies |
|---|---|---|
| **ADAMTS18** | 170692 | 2 |
| **BTG3** | 10950 | 3 |
| **DDX60** | 55601 | 1 |
| **HECW1** | 23072 | 1 |
| **PTPRK** | 5796 | 1 |
| **WWC3** | 7507 | 1 |
