# Supplementary material for: TRIM65 promotes renal cell carcinoma through ubiquitination and degradation of BTG3
Source: Cell Death Dis. 2024 May 22;15(5):355. doi: 10.1038/s41419-024-06741-3 (PMC11111765; doi:10.1038/s41419-024-06741-3)

Full unedited gel for figure

Fig 1 A

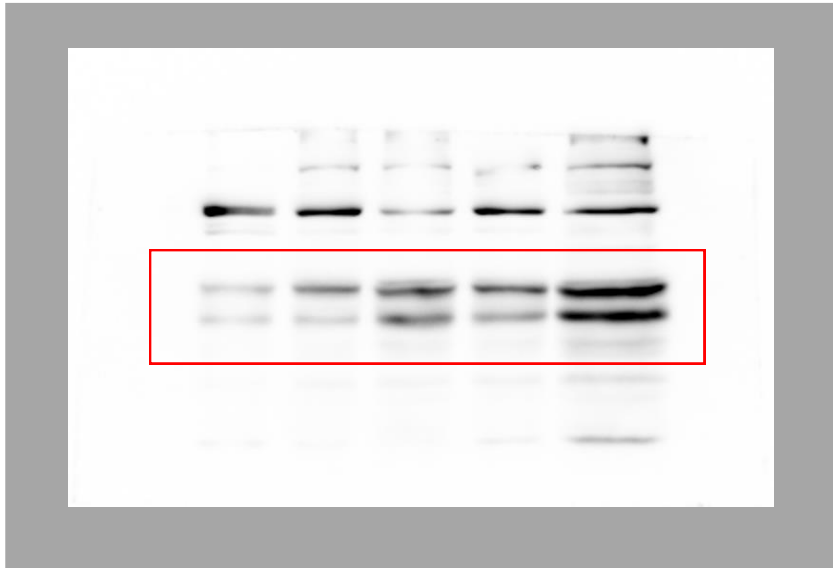

IB:TRIM65

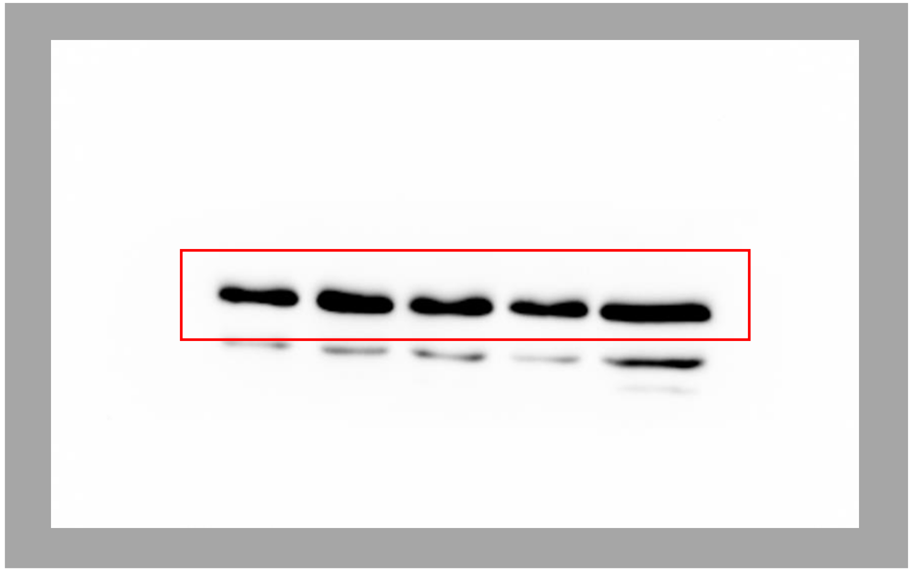

IB:β-actin

Fig 2 E

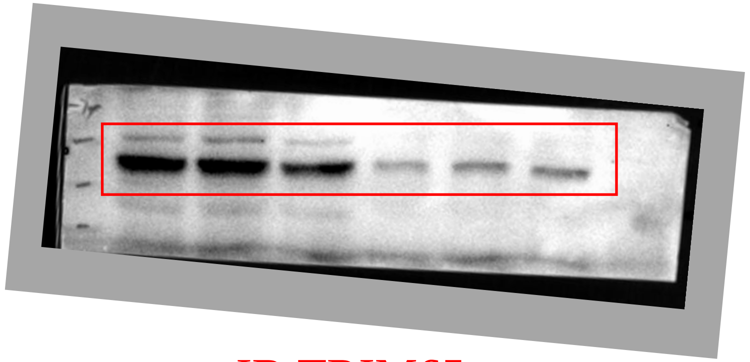

IB:TRIM65

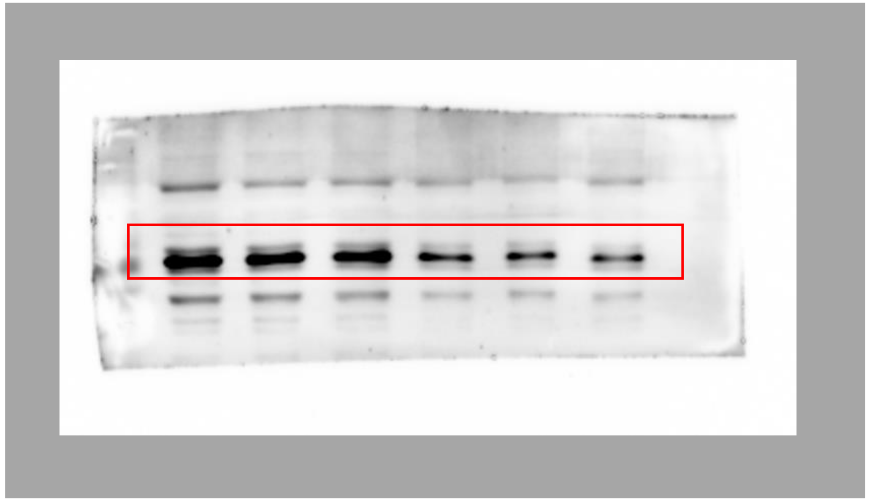

IB:PCNA

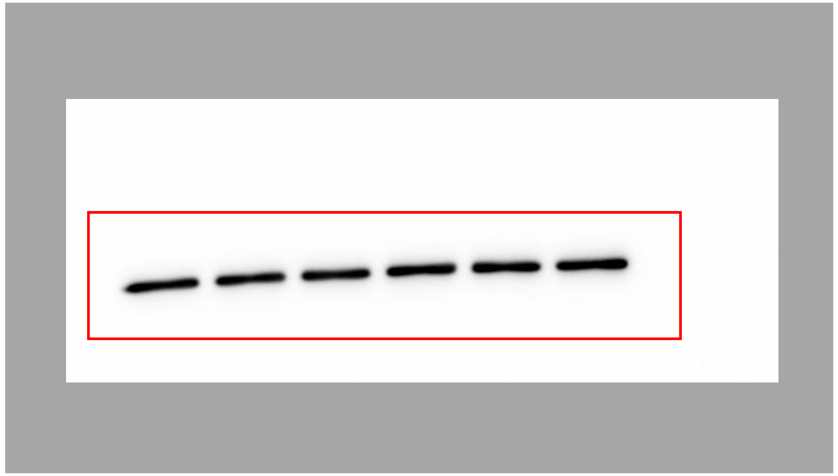

IB:β-actin

Fig 2 J

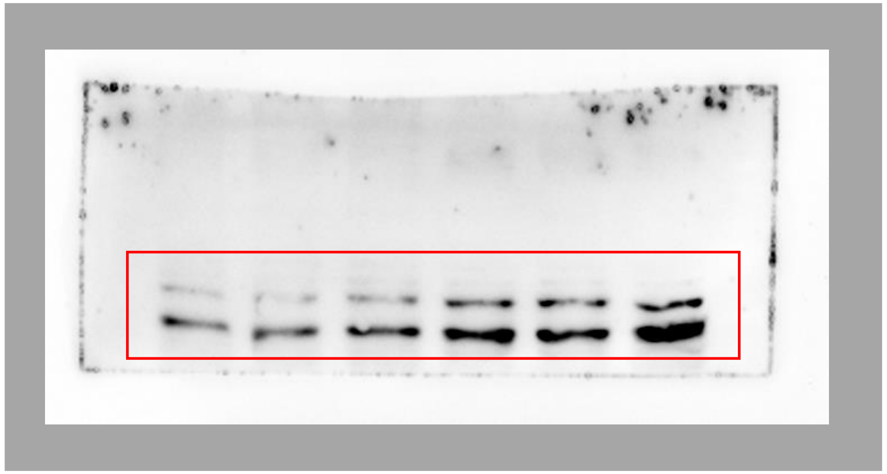

IB:TRIM65

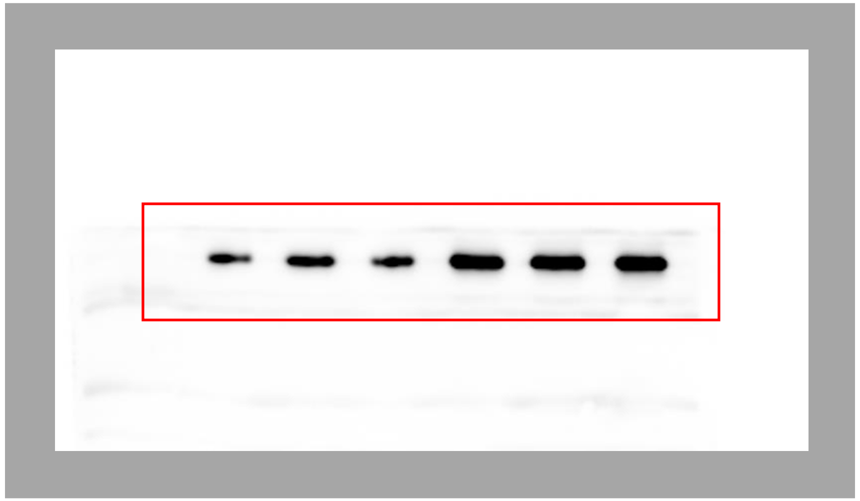

IB:PCNA

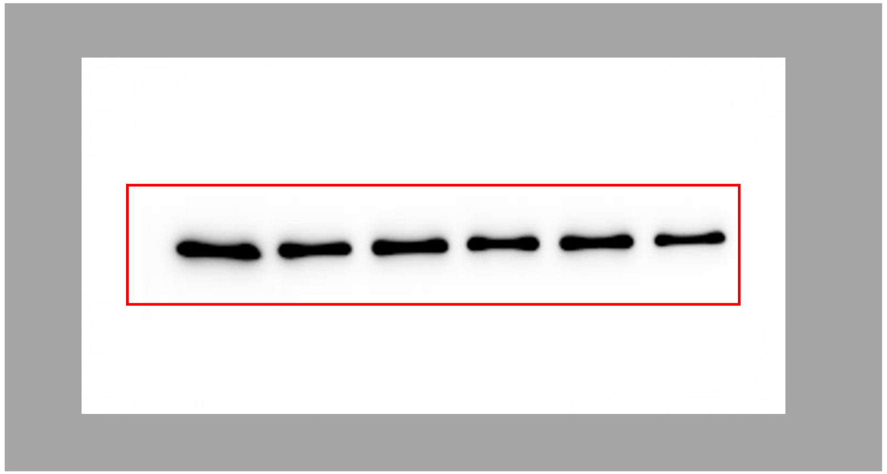

IB:β-actin

Fig 3 B

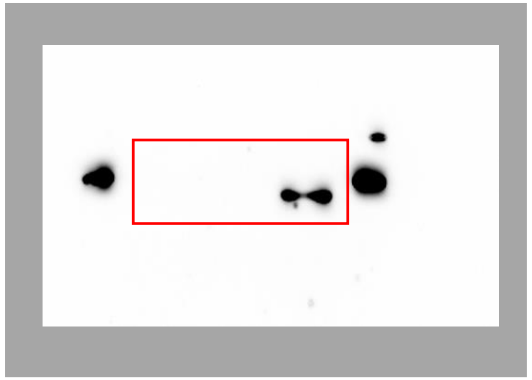

IP:Flag IB:HA

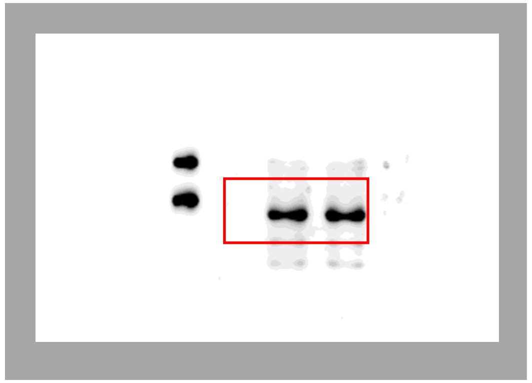

IP:HA IB:HA

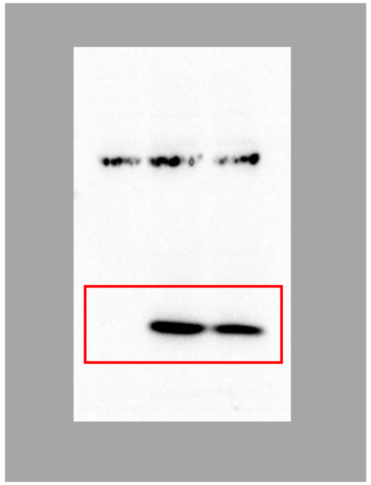

LYSIS IB:HA

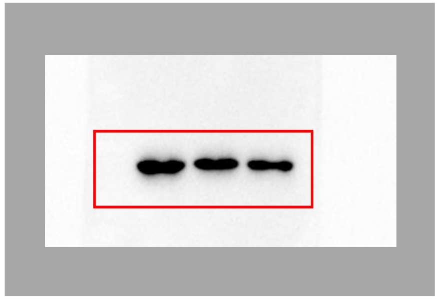

LYSIS IB:GAPDH

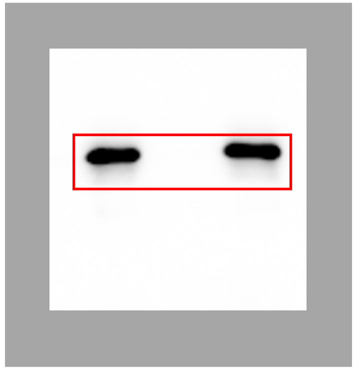

IP:Flag IB:TRIM65

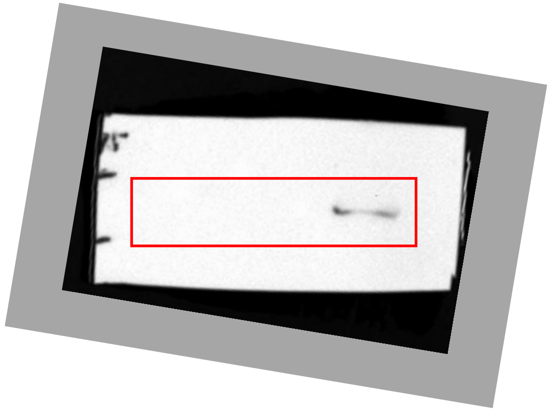

IP:HA IB:TRIM65

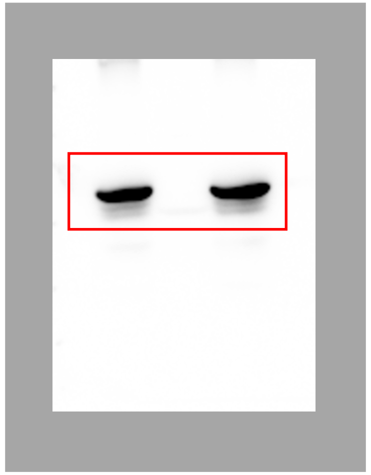

LYSIS IB:TRIM65

Fig 3 C

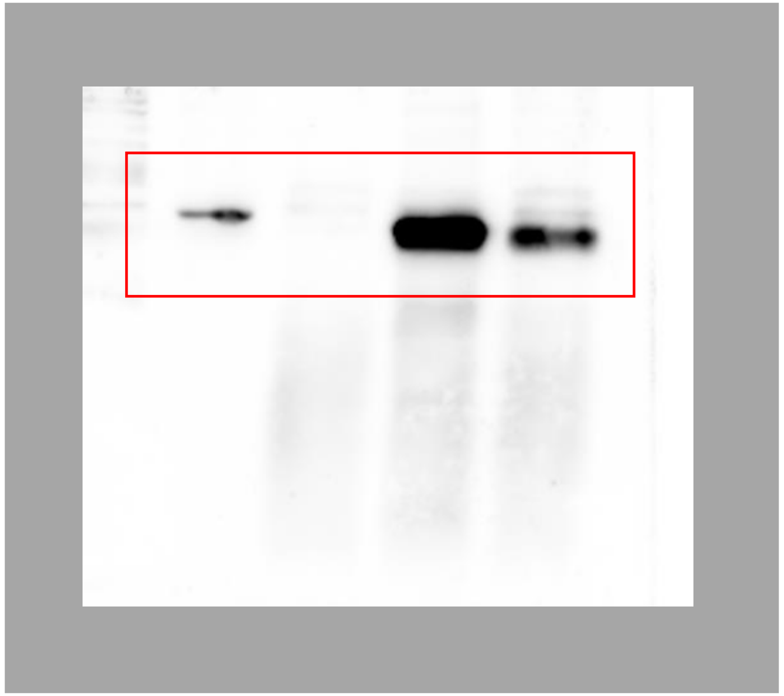

IB:TRIM65

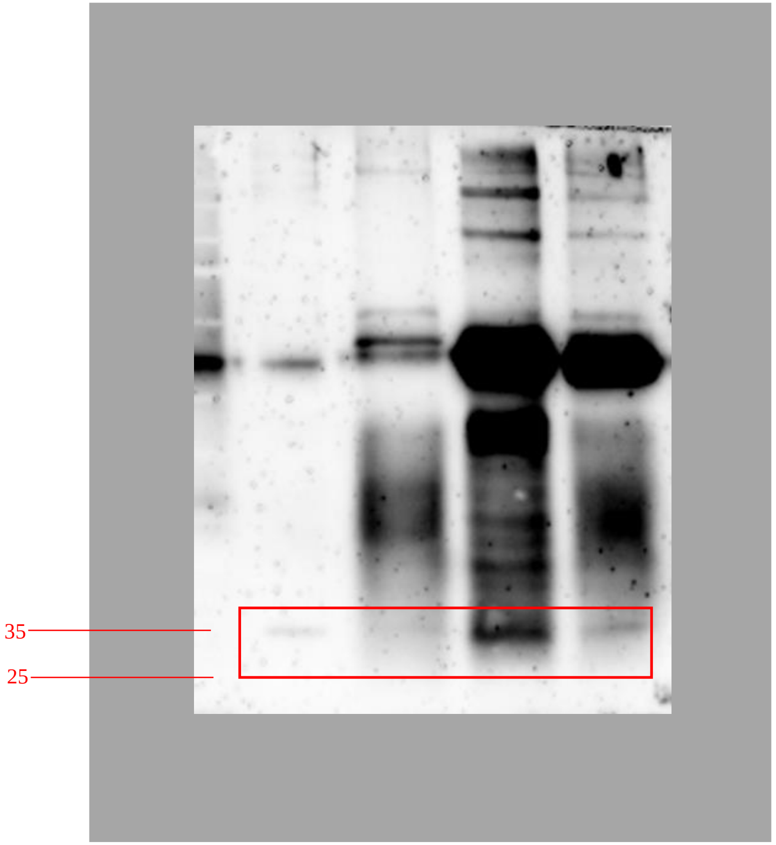

IB:BTG3

Fig 3 D

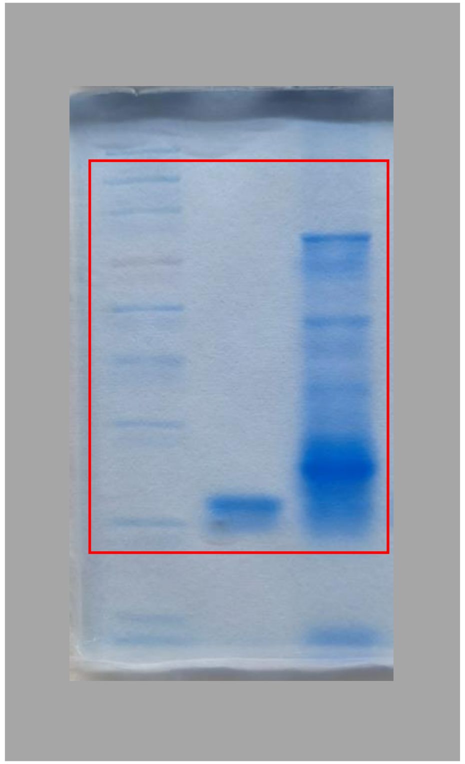

Coomassie brilliant blue

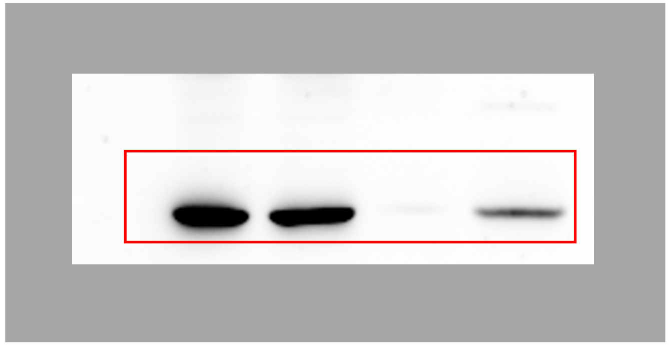

IB:HA

Fig 3 F

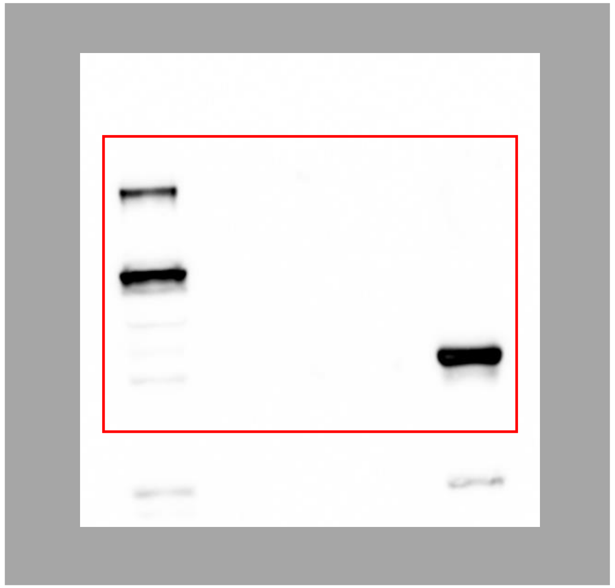

IP IB:GFP

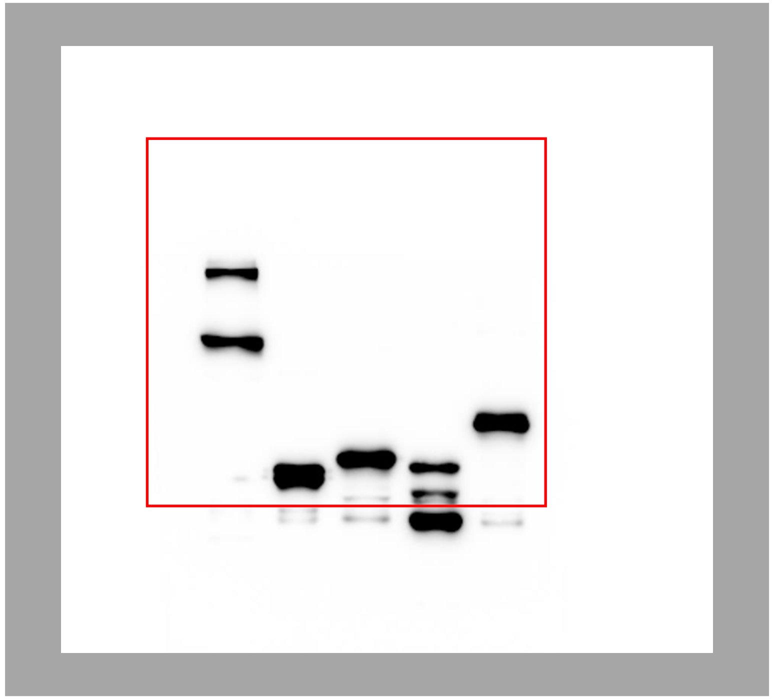

LYSIS IB:GFP

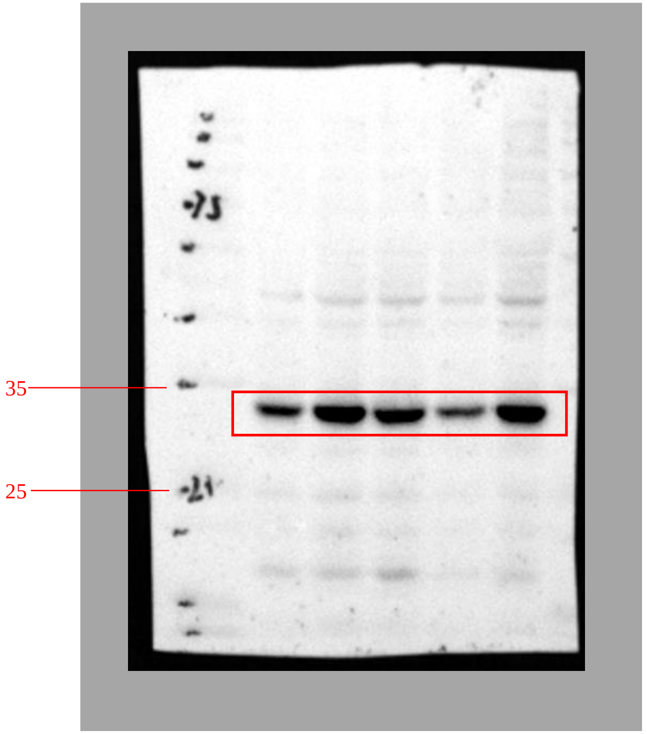

LYSIS IB:BTG3

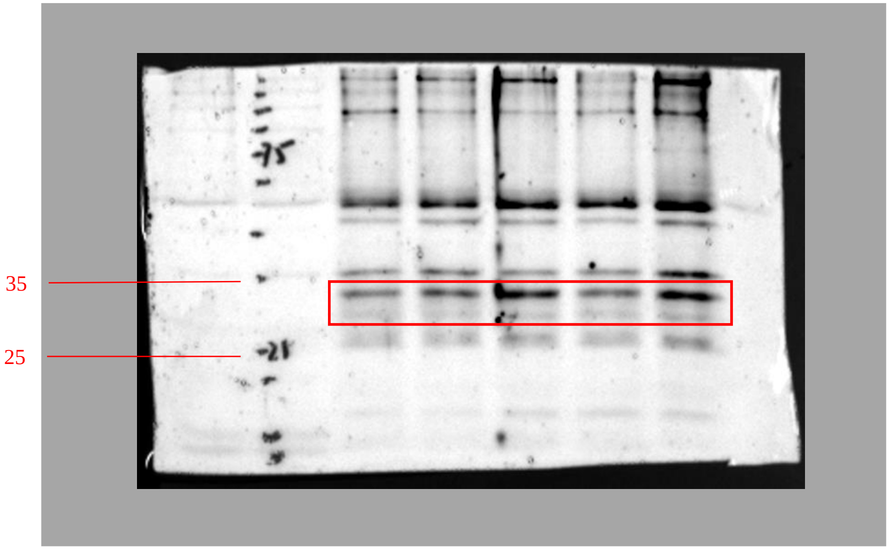

IP IB:BTG3

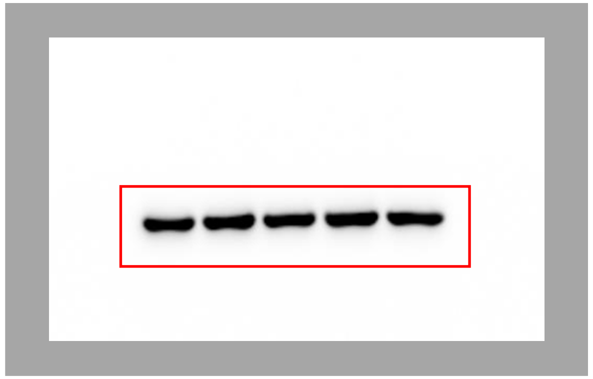

LYSIS IB:β-actin

Fig 3 H

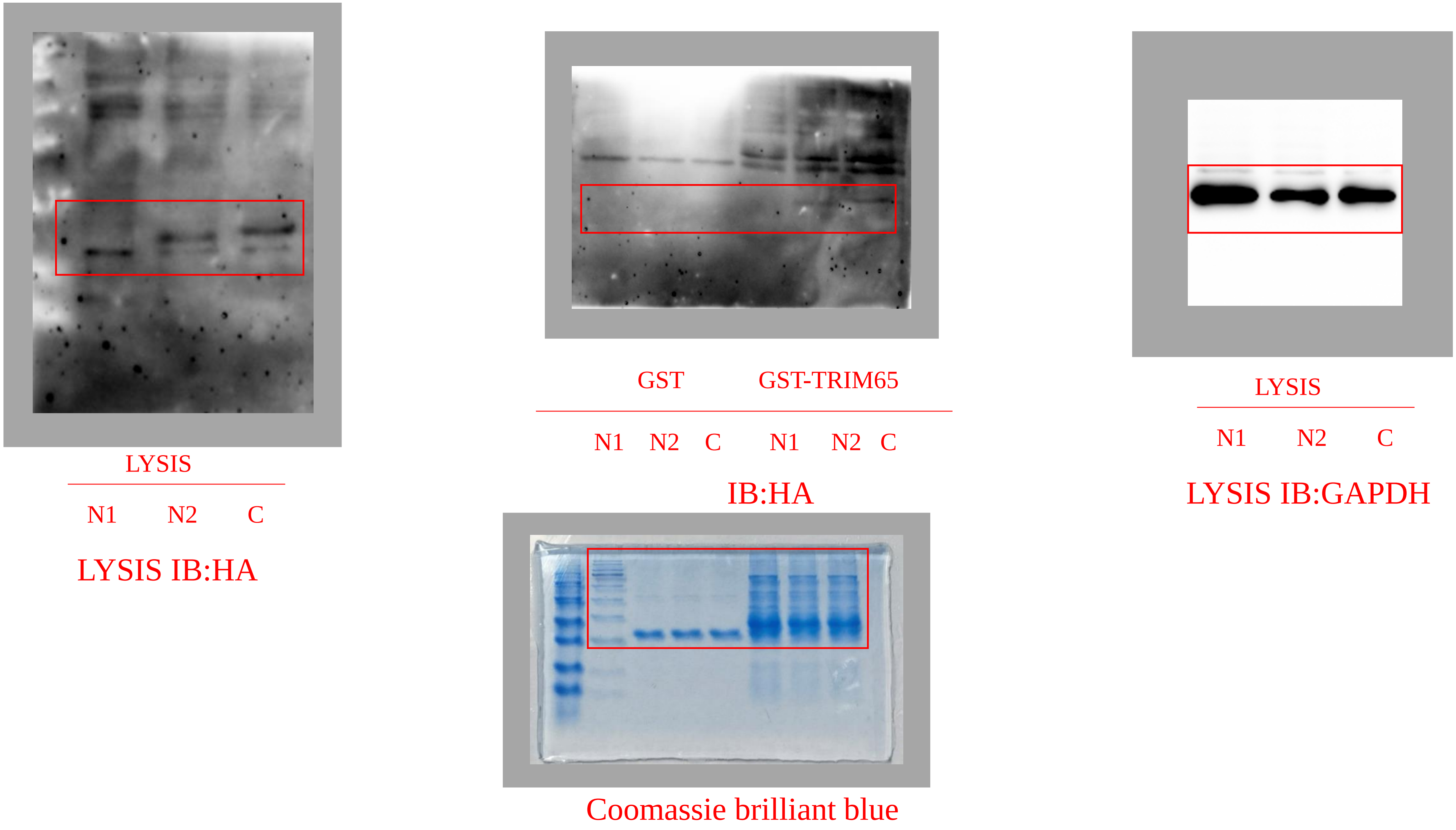
LYSIS
N1 N2 C
LYSIS IB:HA
GST
GST-TRIM65
N1 N2 C N1 N2 C
IB:HA
Coomassie brilliant blue
LYSIS
N1 N2 C
LYSIS IB:GAPDH

Fig 4 A

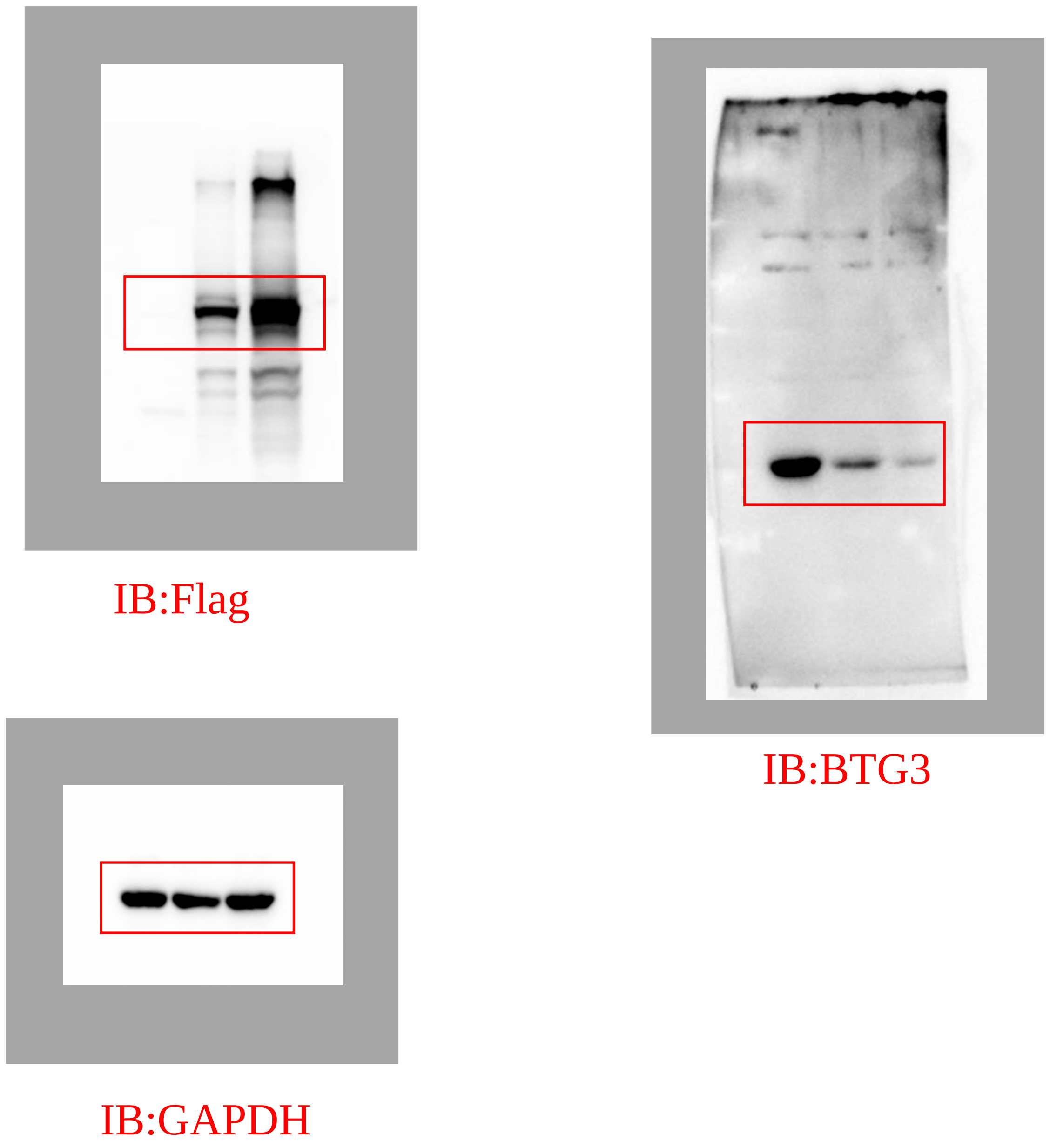

Fig 4 B

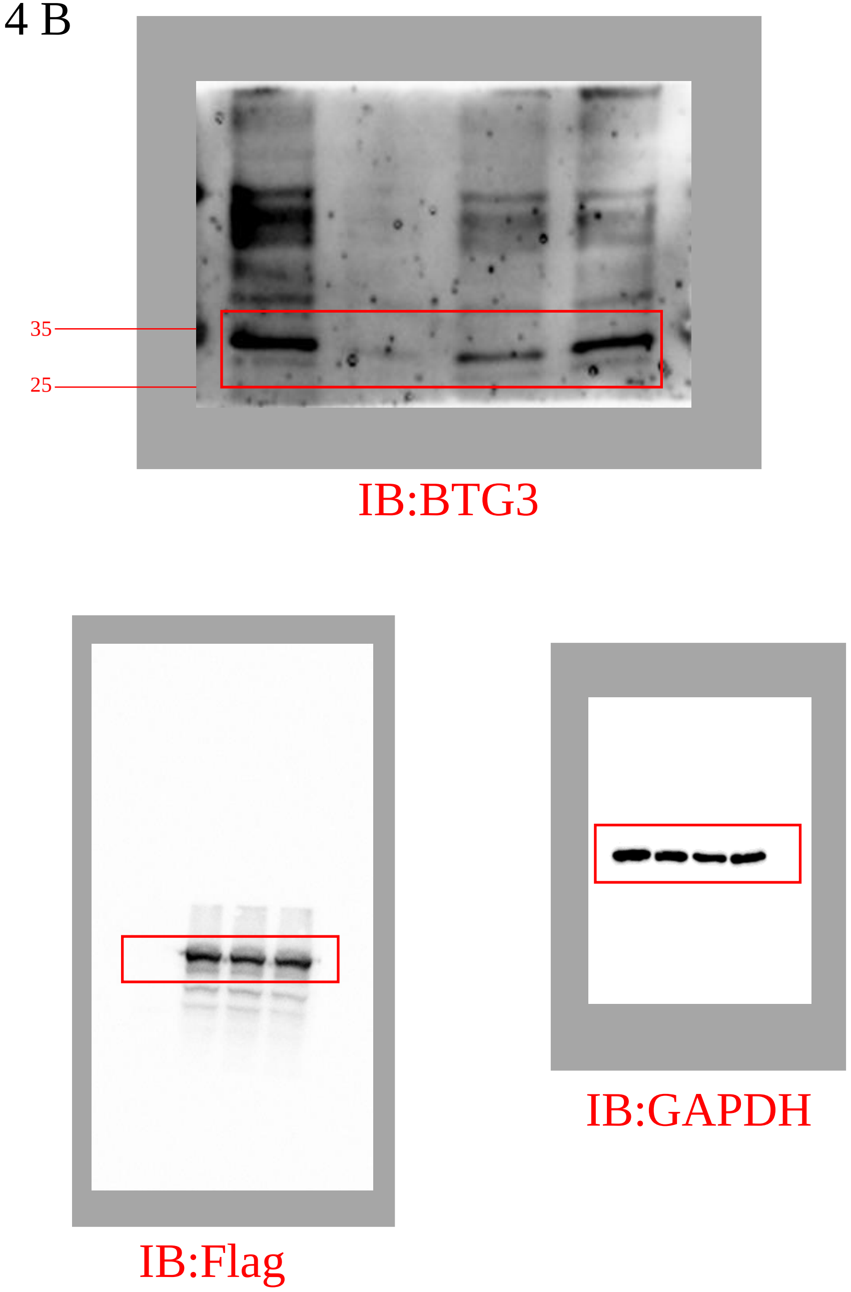

Fig 4 C

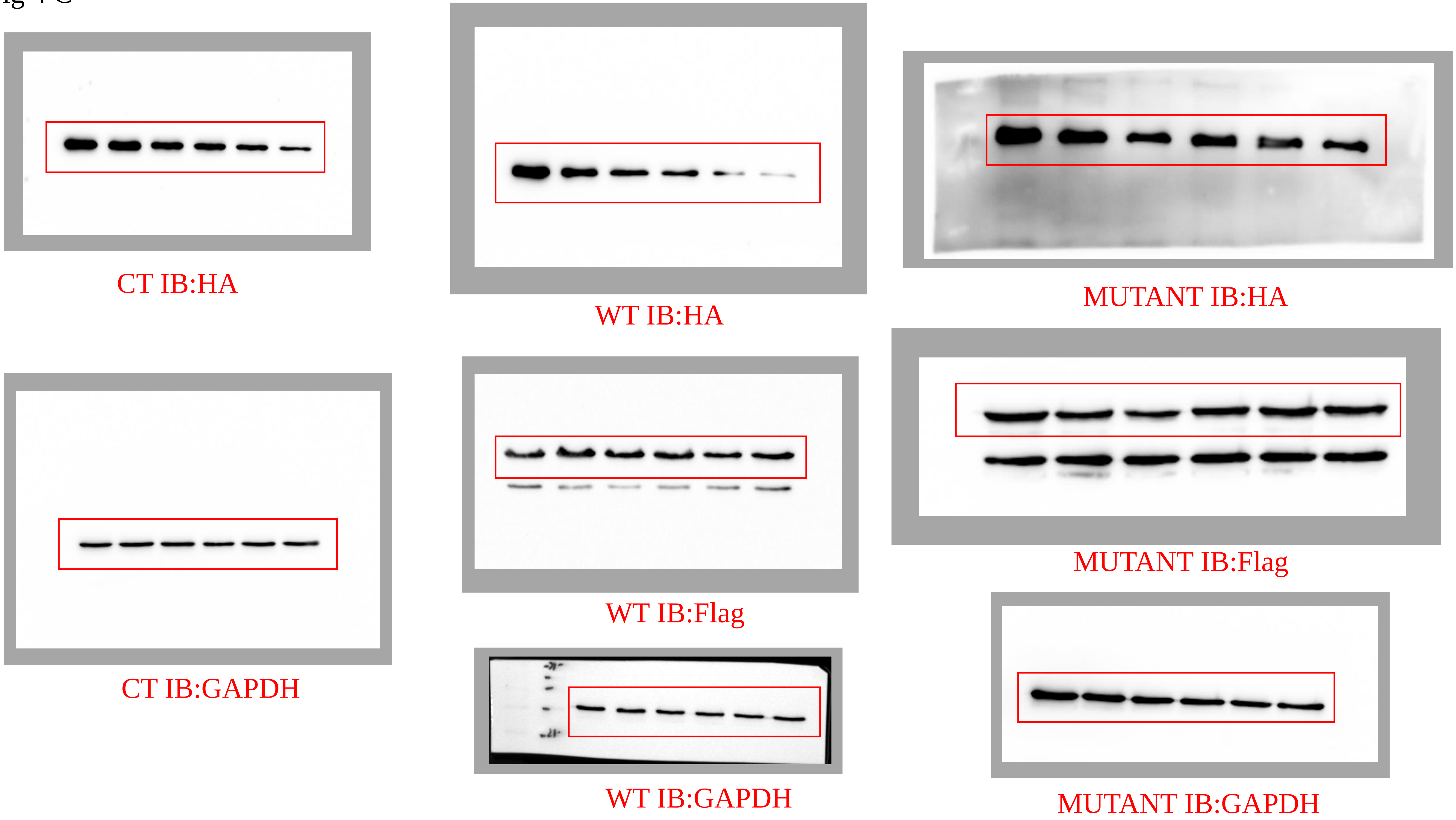
CT IB:HA
WT IB:HA
MUTANT IB:HA
CT IB:GAPDH
WT IB:Flag
MUTANT IB:Flag
WT IB:GAPDH
MUTANT IB:GAPDH

Fig 4 D

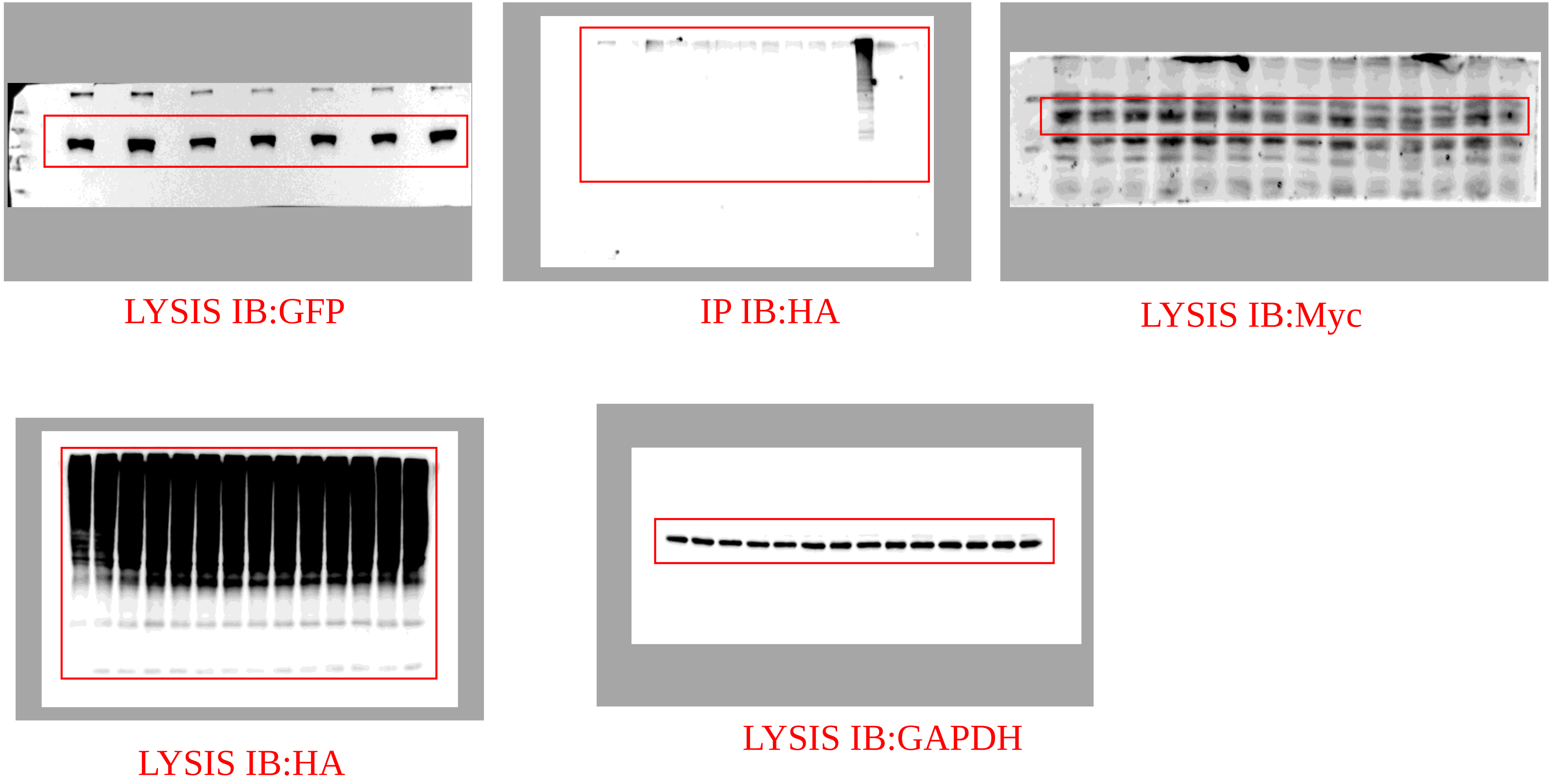

Fig 4 E

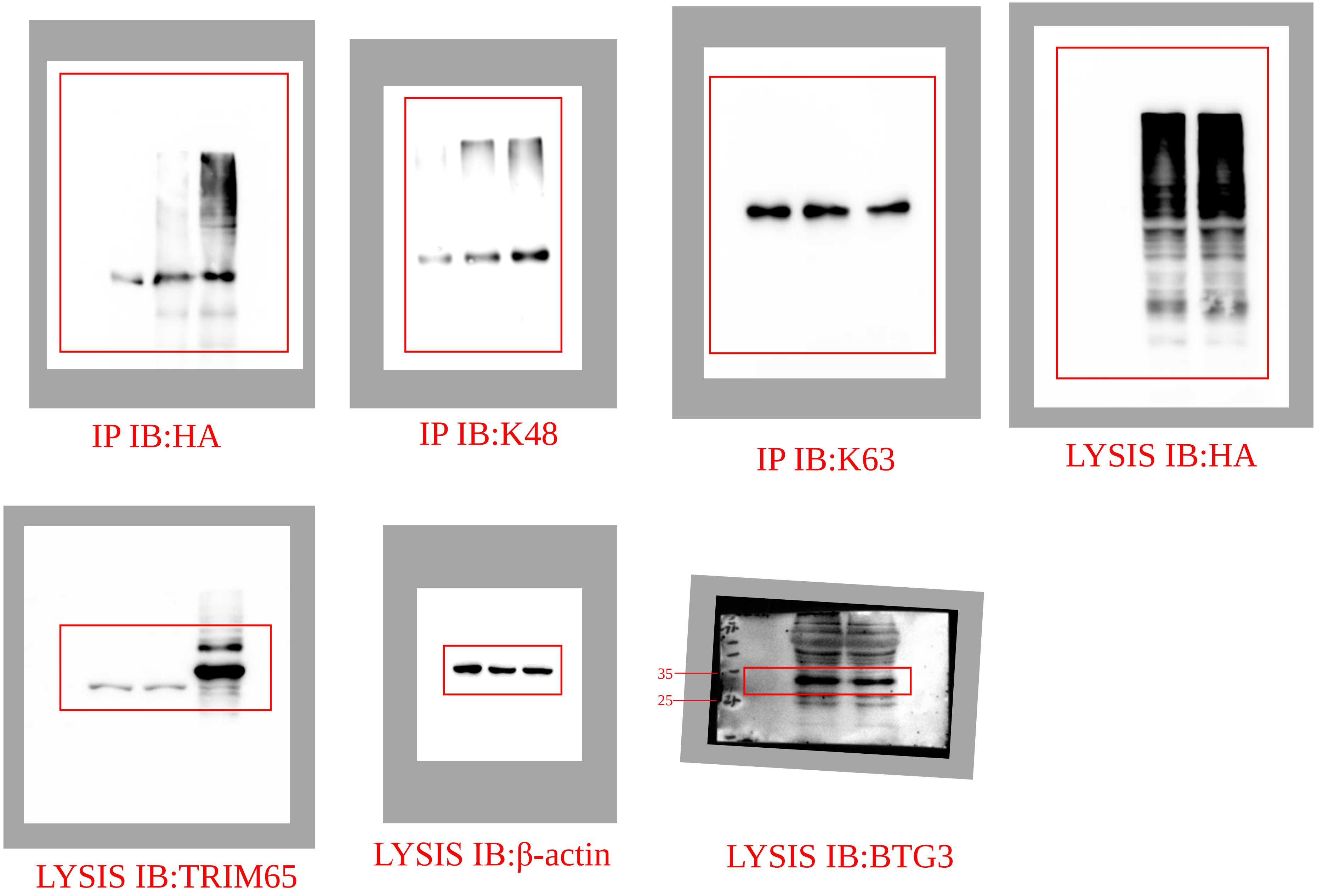

IP IB:HA
IP IB:K48
IP IB:K63
LYSIS IB:HA
LYSIS IB:TRIM65
LYSIS IB:β-actin
35
25
LYSIS IB:BTG3

Fig 4 F

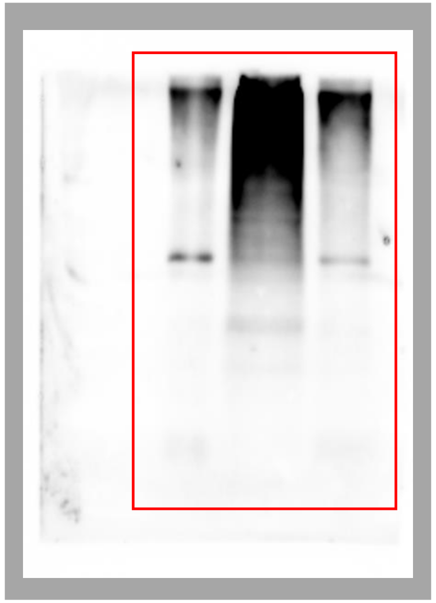

IP IB:HA

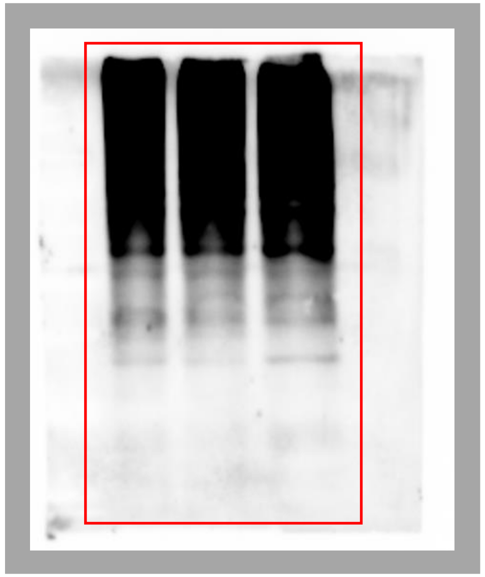

LYSIS IB:HA

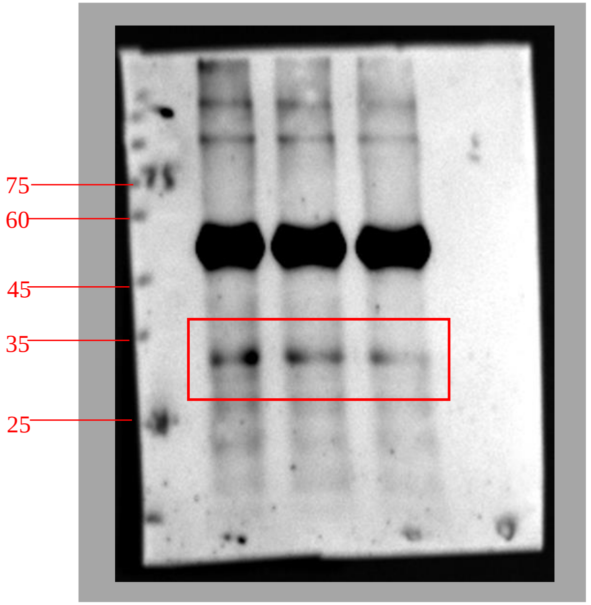

LYSIS IB:BTG3

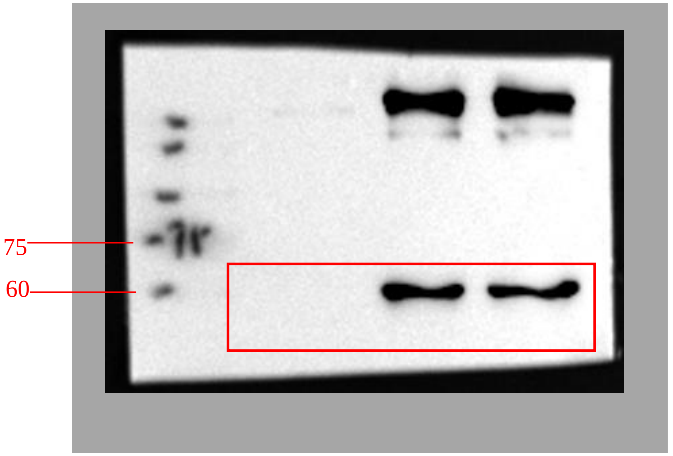

LYSIS IB:Flag

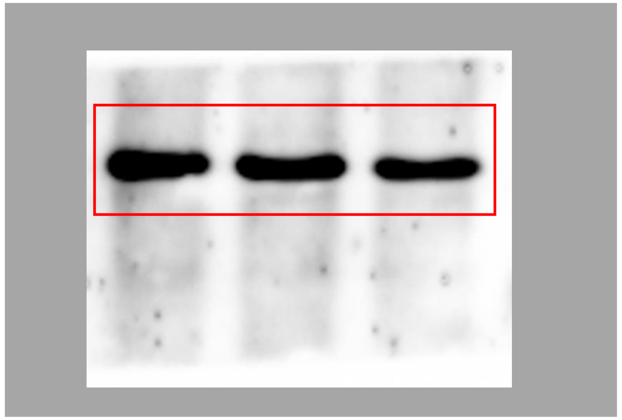

LYSIS IB:GAPDH

Fig 4 G

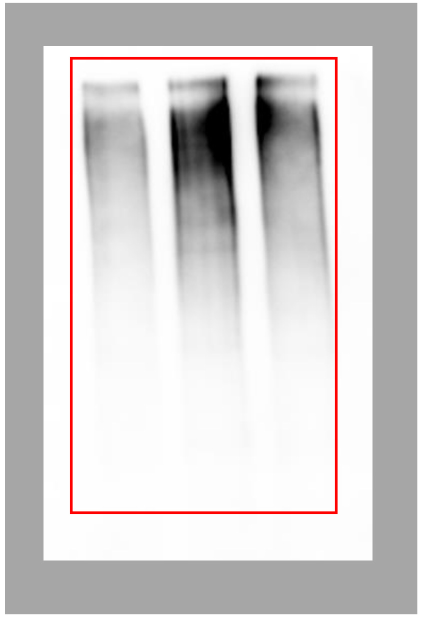

IP IB:HA

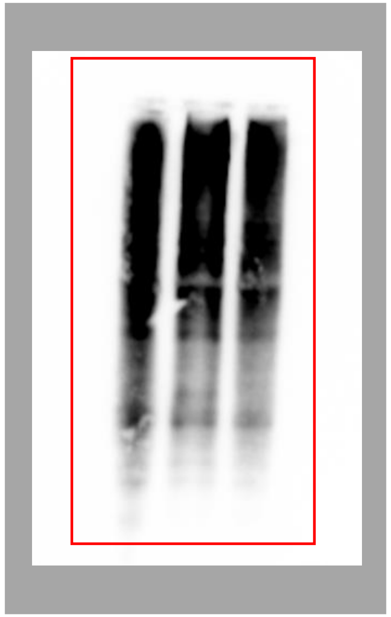

LYSIS IB:HA

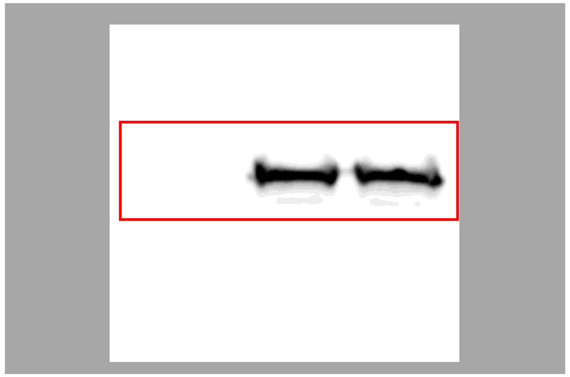

LYSIS IB:Flag

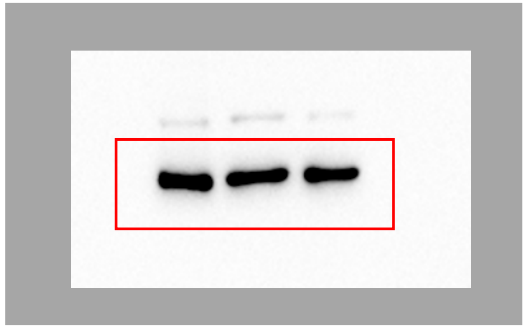

LYSIS IB:GAPDH

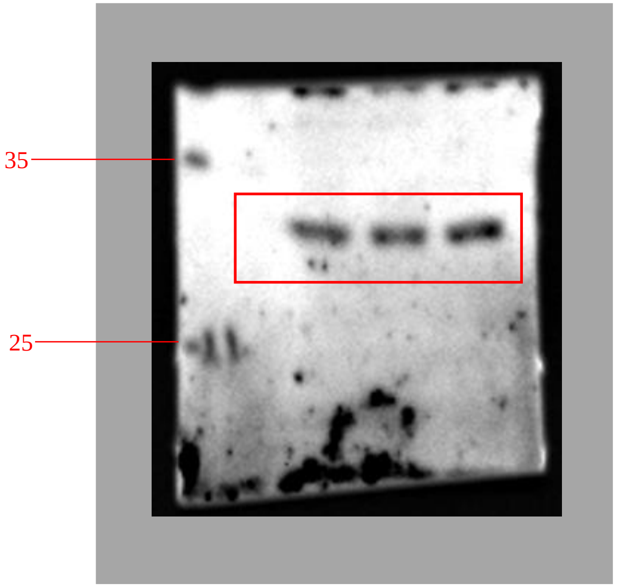

LYSIS IB:BTG3

Fig 4 H

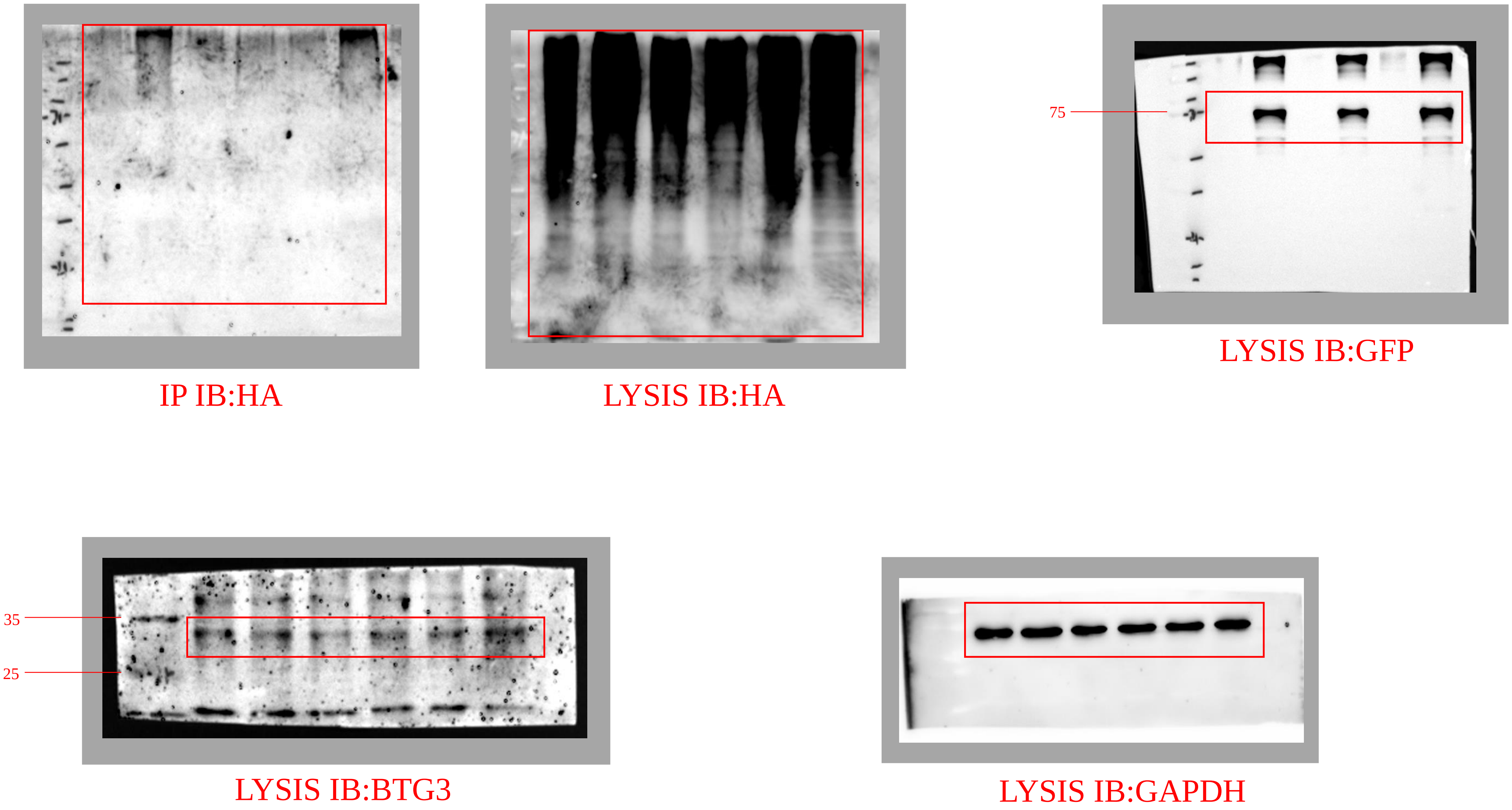
IP IB:HA
LYSIS IB:HA
75
LYSIS IB:GFP
35
25
LYSIS IB:BTG3
LYSIS IB:GAPDH

Fig 4 I

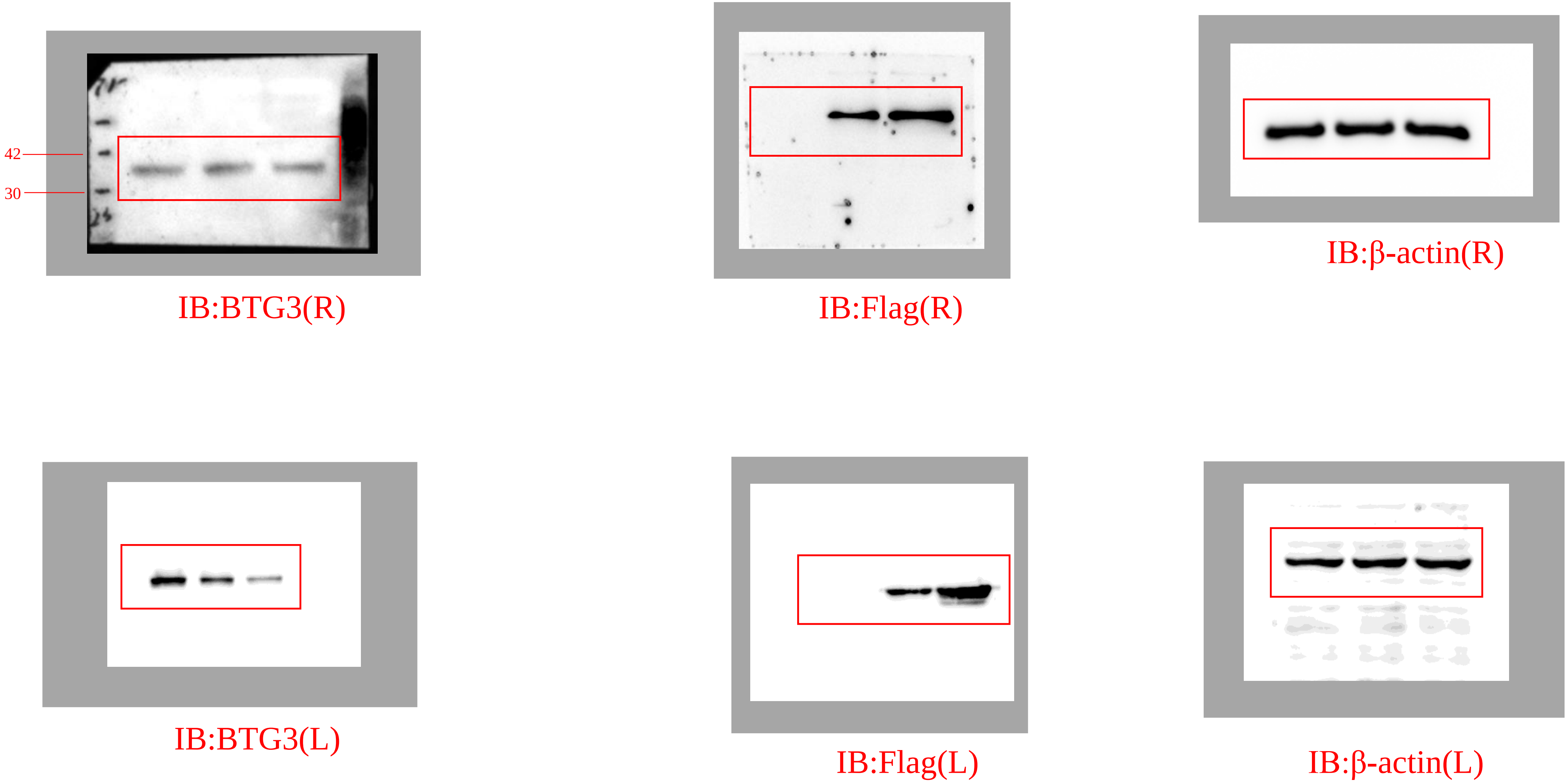
42
30
IB:BTG3(R)
IB:Flag(R)
IB:β-actin(R)
IB:BTG3(L)
IB:Flag(L)
IB:β-actin(L)

Fig 4 J

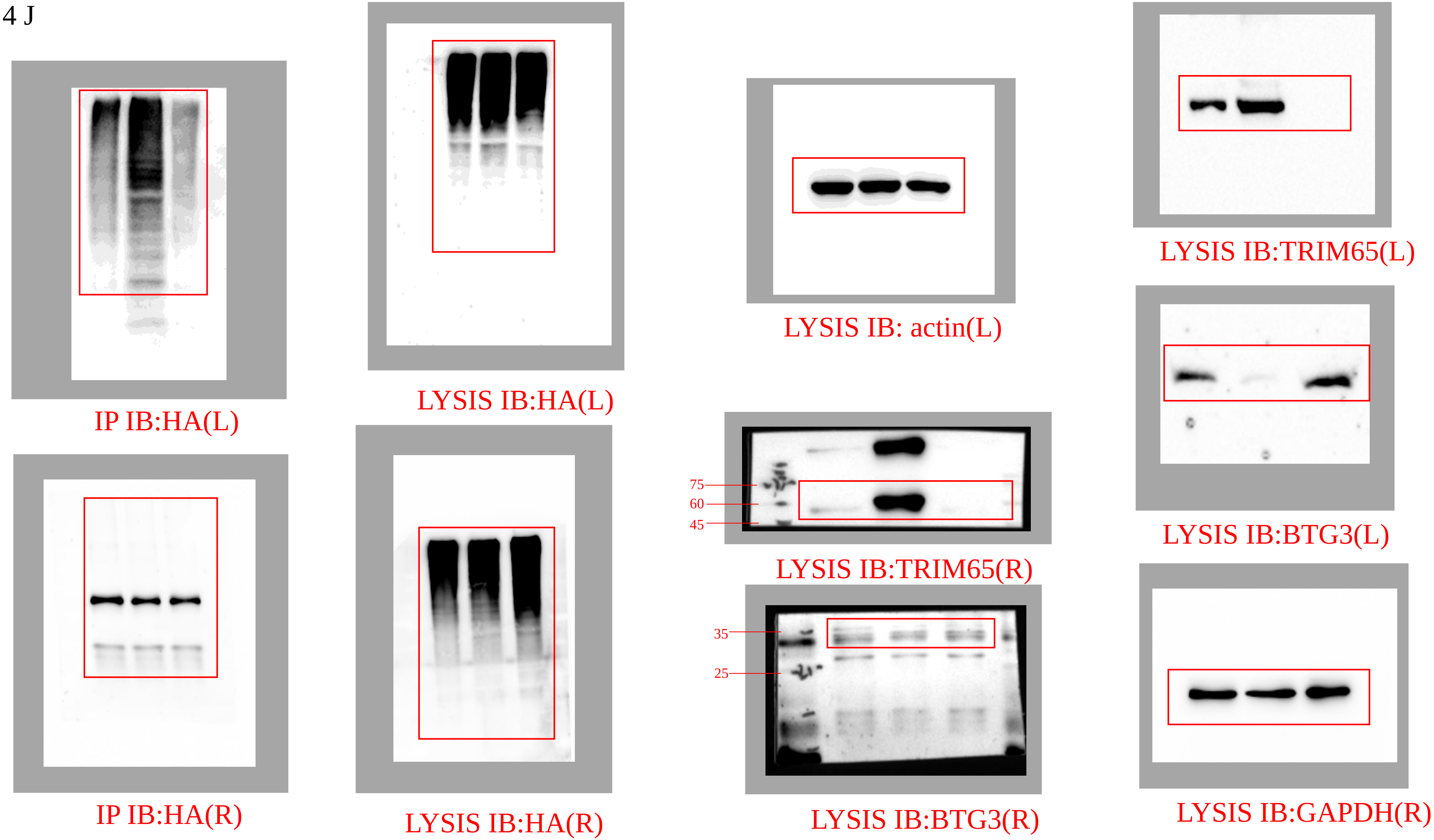
IP IB:HA(L)
LYSIS IB:HA(L)
LYSIS IB: actin(L)
LYSIS IB:TRIM65(L)
LYSIS IB:BTG3(L)
IP IB:HA(R)
LYSIS IB:HA(R)
75
60
45
LYSIS IB:TRIM65(R)
35
25
LYSIS IB:BTG3(R)
LYSIS IB:GAPDH(R)

Fig 5 E

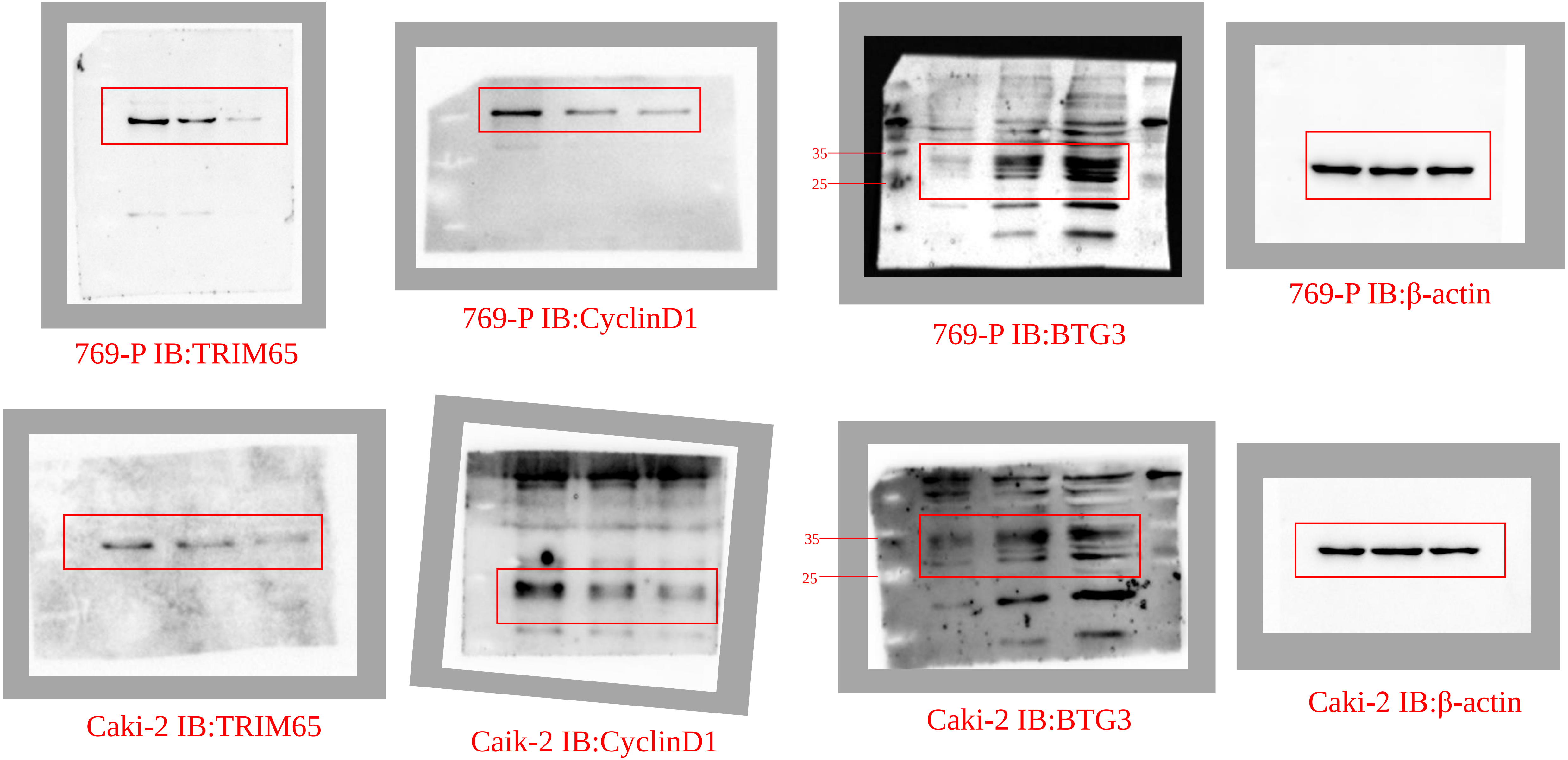

769-P IB:TRIM65
769-P IB:CyclinD1
35
25
769-P IB:BTG3
769-P IB:β-actin
Caki-2 IB:TRIM65
Caik-2 IB:CyclinD1
35
25
Caki-2 IB:BTG3
Caki-2 IB:β-actin

Fig 5 F

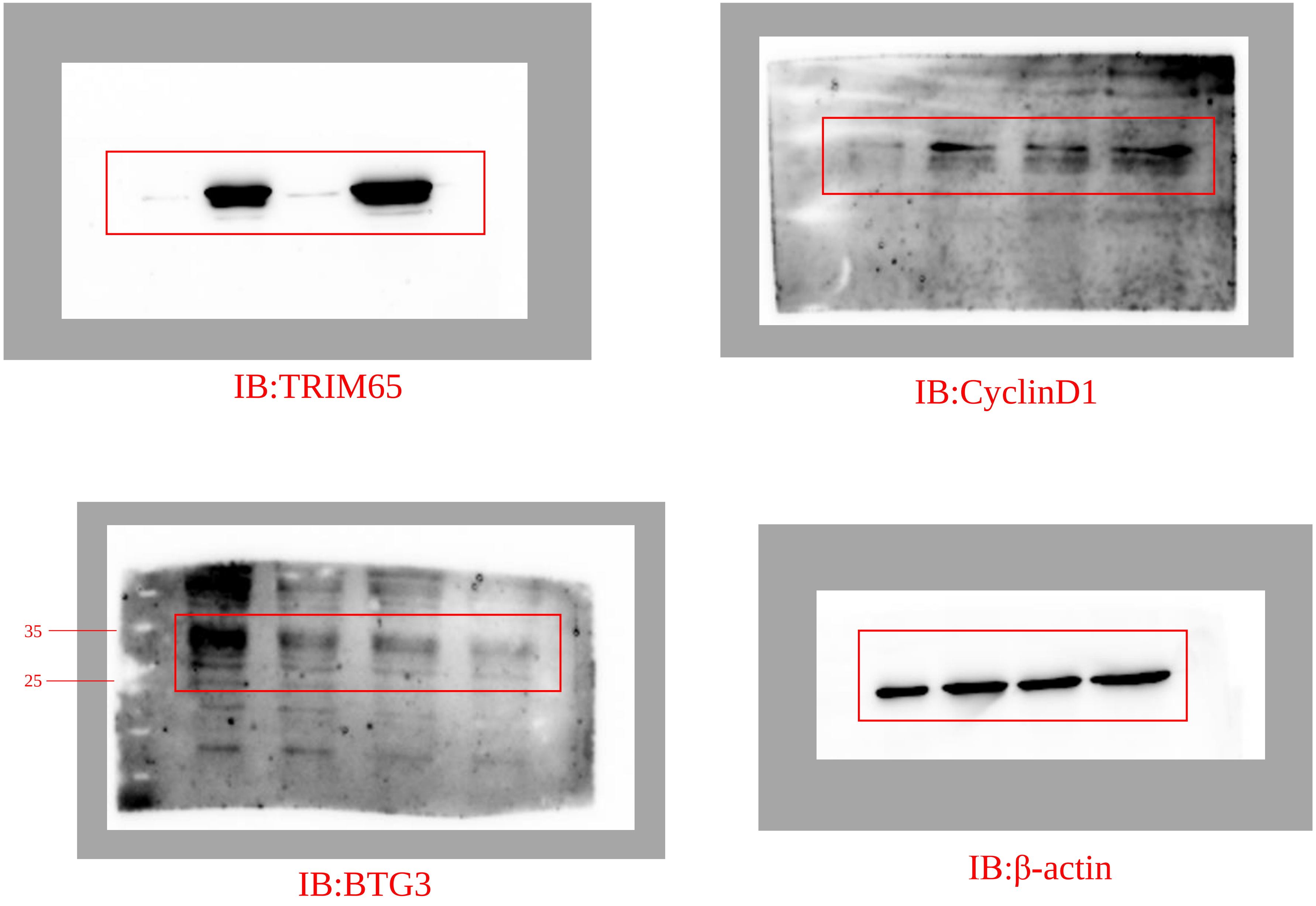

Fig 5 G

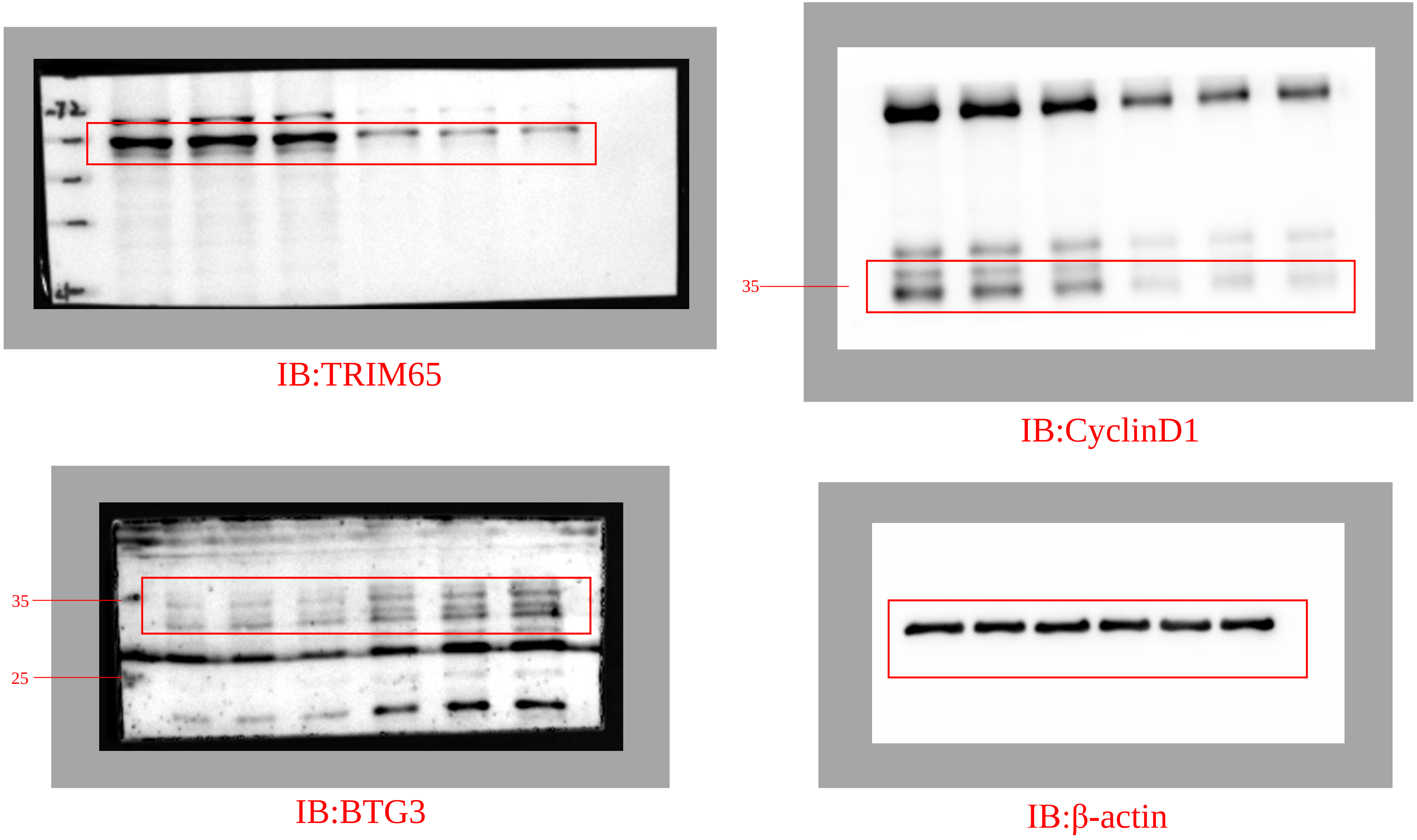

Fig 5 H

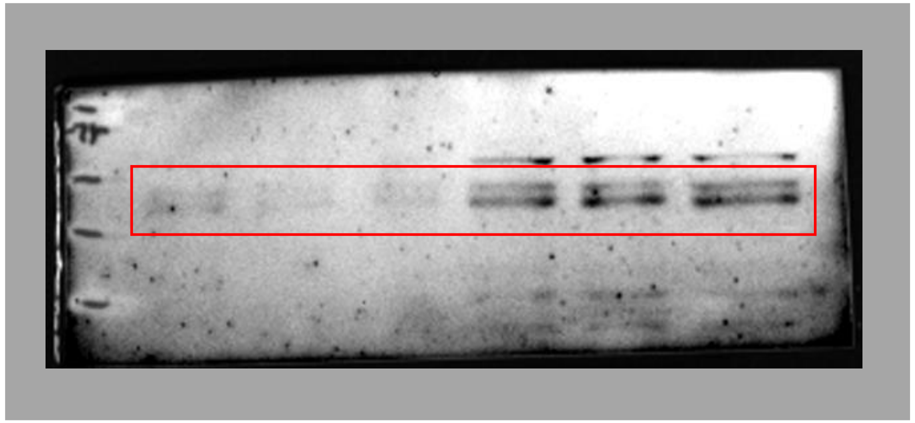

IB:TRIM65

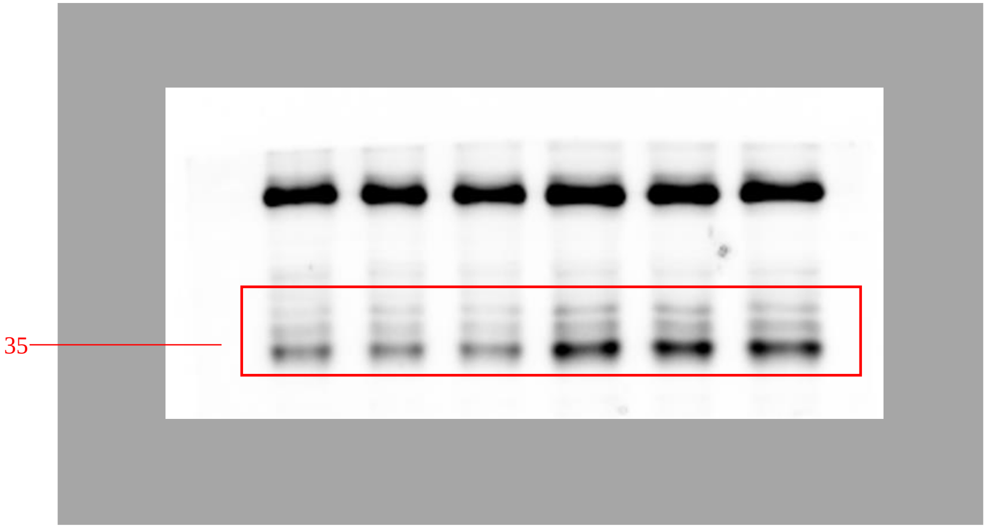

IB:CyclinD1

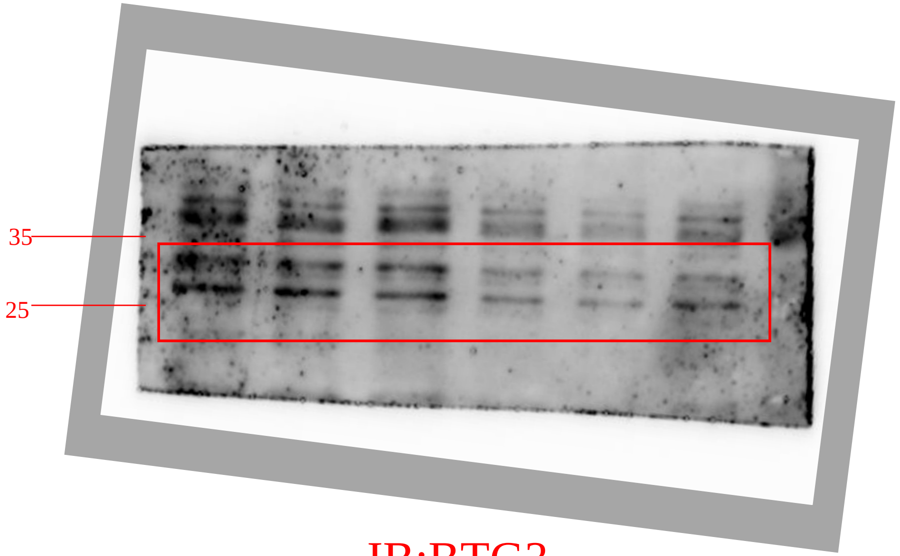

IB:BTG3

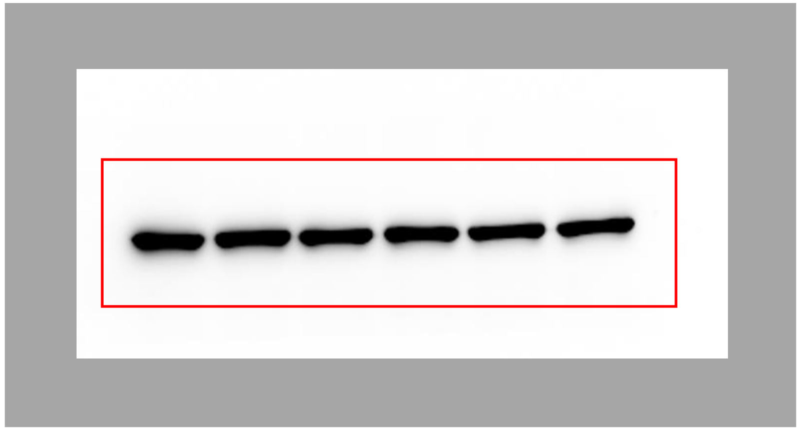

IB:β-actin

Fig 6 A

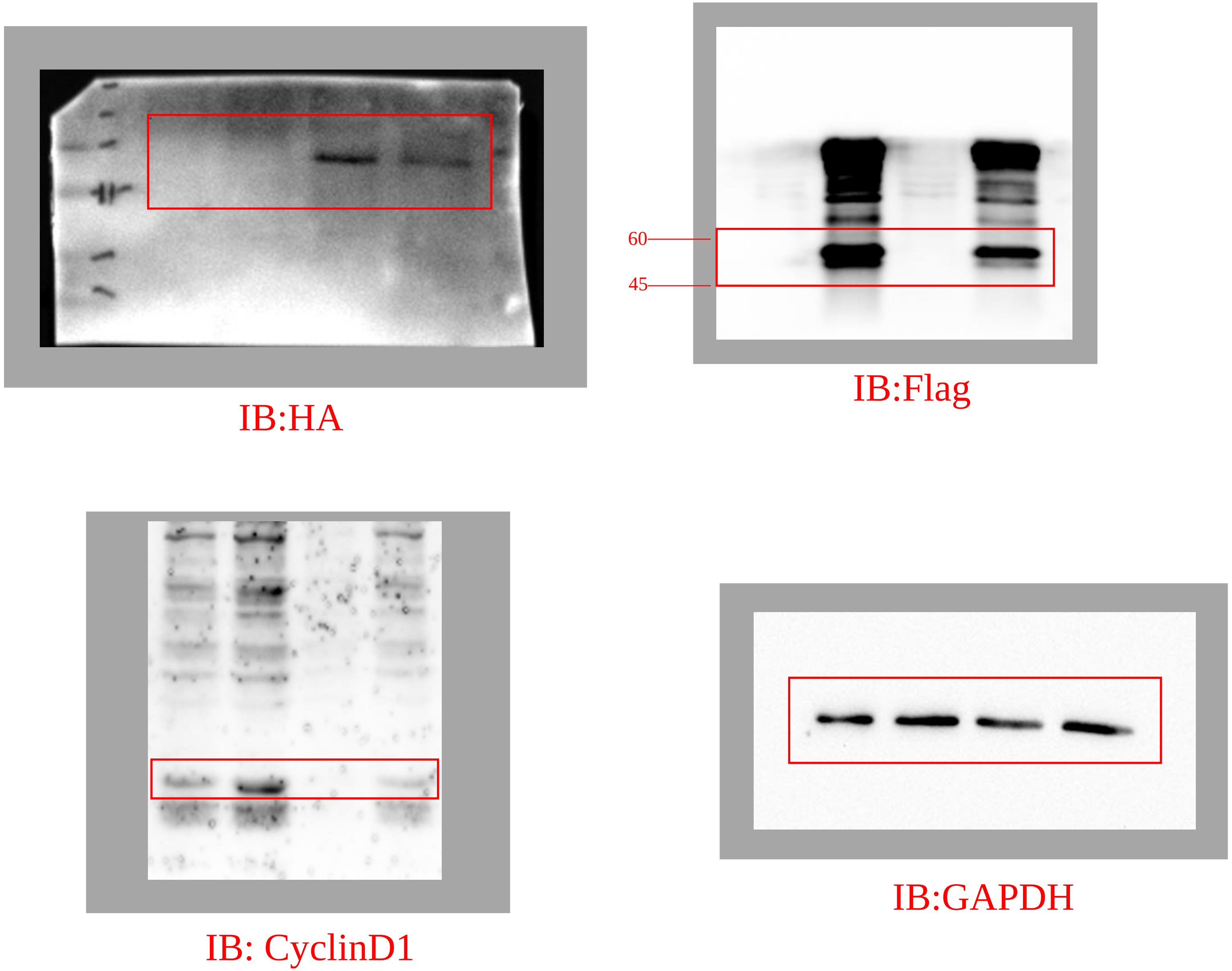

Fig 7 A

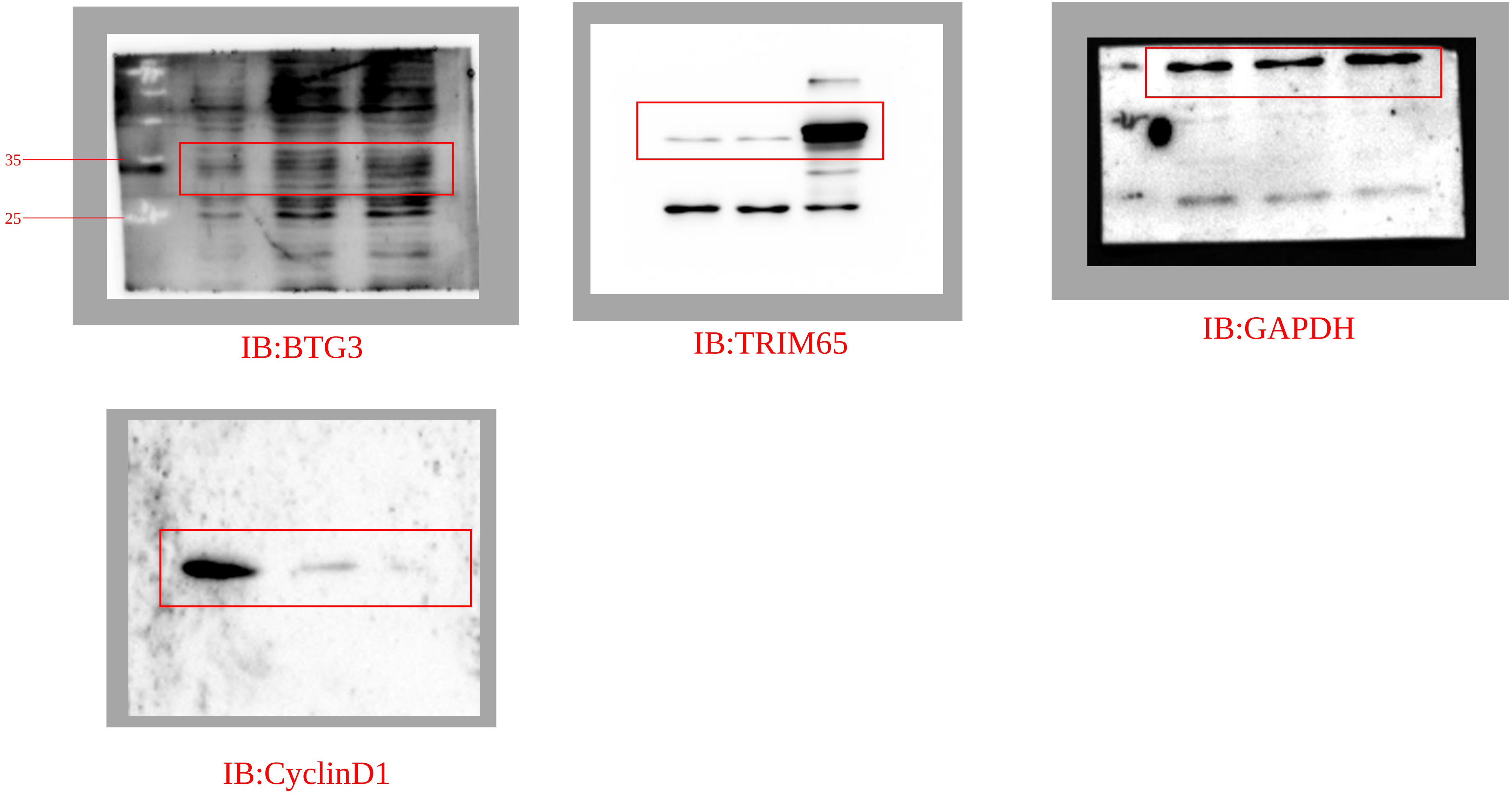

Supplement: Supplementary file 2 — Uncropped WB files [file 41419_2024_6741_MOESM2_ESM.pdf]
